# Transcriptomic and methylation analysis of susceptible and tolerant grapevine genotypes following *Plasmopara viticola* infection

**DOI:** 10.1111/ppl.13771

**Published:** 2022-09-21

**Authors:** Vanessa Azevedo, Loretta Daddiego, Maria Francesca Cardone, Giorgio Perrella, Lisete Sousa, Rita B. Santos, Rui Malhó, Carlo Bergamini, Antonio Domenico Marsico, Andreia Figueiredo, Fiammetta Alagna

**Affiliations:** ^1^ Faculdade de Ciências, Plant Biology Department, Biosystems & Integrative Sciences Institute (BioISI) Universidade de Lisboa Lisbon Portugal; ^2^ Energy Technologies and Renewable Sources Department National Agency for New Technologies, Energy and Sustainable Economic Development (ENEA), Trisaia Research Centre Rotondella Matera Italy; ^3^ Research Centre for Viticulture and Enology Council for Agricultural Research and Economics (CREA) Turi Bari Italy; ^4^ Department of Biosciences University of Milan Milan Italy; ^5^ Department of Statistics and Operations Research, Faculdade de Ciências; Centre of Statistics and its Applications (CEAUL) Universidade de Lisboa Lisbon Portugal

## Abstract

Downy mildew, caused by the biotrophic oomycete *Plasmopara viticola*, is one of the most economically significant grapevine diseases worldwide. Current strategies to cope with this threat rely on the massive use of chemical compounds during each cultivation season. The economic costs and negative environmental impact associated with these applications increased the urge to search for sustainable strategies of disease control. Improved knowledge of plant mechanisms to counteract pathogen infection may allow the development of alternative strategies for plant protection. Epigenetic regulation, in particular DNA methylation, is emerging as a key factor in the context of plant–pathogen interactions associated with the expression modulation of defence genes. To improve our understanding of the genetic and epigenetic mechanisms underpinning grapevine response to *P. viticola*, we studied the modulation of both 5‐mC methylation and gene expression at 6 and 24 h post‐infection (hpi). Leaves of two table grape genotypes (*Vitis vinifera*), selected by breeding activities for their contrasting level of susceptibility to the pathogen, were analysed. Following pathogen infection, we found variations in the 5‐mC methylation level and the gene expression profile. The results indicate a genotype‐specific response to pathogen infection. The tolerant genotype (N23/018) at 6 hpi exhibits a lower methylation level compared to the susceptible one (N20/020), and it shows an early modulation (at 6 hpi) of defence and epigenetic‐related genes during *P. viticola* infection. These data suggest that the timing of response is an important mechanism to efficiently counteract the pathogen attack.

## 1 INTRODUCTION

Viticulture is one of the most important agricultural activities worldwide. In 2020, 7.3 million hectares were estimated to be cultivated as vineyards (OIV, 2021 ). The cultivated *Vitis vinifera* L. is highly susceptible to downy mildew, caused by the obligatory oomycete *Plasmopara viticola* (Berk. & Curtis) Berl. & De Toni, which significantly influences the plant lifespan, fruit quality and production quantity (Armijo et al., 2016; Boso et al., 2014; Brilli et al., 2018; Buonassisi et al., 2017; Eisenmann et al., 2019; Vezzulli et al., 2018). High amounts of pesticides are used during the growing season to control this disease, harbouring both environmental and health impacts. To promote a more sustainable approach to control this disease, efforts have been conducted to expand the knowledge of this interaction and breeding new cultivars resistant to *P. viticola* have become a promising approach. Therefore, a deeper knowledge of grapevine defence mechanisms is crucial to identify candidates for resistance introgression.

The grapevine defence system relies on intricate communication between host and pathogen, leading to broad physiological modulation and the activation of molecular layers of defence. Previous studies have shown that a broad modulation of transcripts, proteins, lipids and metabolites occurs in the first hours of interaction between grapevine and *P. viticola* (Buonassisi et al., 2017; Figueiredo et al., 2017, 2018; Fröbel & Zyprian, 2019; Laureano et al., 2018; Liu et al., 2020; Maia et al., 2019; Nascimento et al., 2019; Santos et al., 2020). Upon being challenged with biotic or abiotic stresses, plants can create a “memory” of the response obtained. This response is known as defence priming, and it can also be mediated by different epigenetic mechanisms (Espinas et al., 2016; Lämke & Bäurle, 2017; Laurell et al., 2022; Ramirez‐Prado, Abulfaraj, et al., 2018). Epigenetic modulation is known to regulate gene expression that leads to phenotypic plasticity aiming at plant adaptation and survival to external stimuli (Gallusci et al., 2017; Ramos‐Cruz et al., 2021; Xie et al., 2017; Yan et al., 2019). Epigenetic modifications may affect plant defence at a long‐term level contributing to a transgenerational inheritable defence strategy (Espinas et al., 2016; Lämke & Bäurle, 2017; Laurell et al., 2022; Ramirez‐Prado, Abulfaraj, et al., 2018; Xie et al., 2017).

In the context of plant–pathogen interactions, epigenetic modulation has been shown to impact the outcome of the host defence. Several mechanisms leading to epigenetic modifications have been described so far, including DNA methylation, chromatin rearrangement, histone modifications and the establishment of gene silencing through RNA interference (RNAi) (Barozai & Aziz, 2018; Espinas et al., 2016; Hoang et al., 2018; Ramirez‐Prado, Piquerez, et al., 2018; Ramos‐Cruz et al., 2021; Yan et al., 2019). Several studies on DNA methylation have been performed on different types of host–pathogens interaction (Huang & Jin, 2022). As an adaptive strategy to biotic stresses, plant DNA methylation may lead to a broad transcriptome reprogramming since the pressure on the different positions of the cytosine methylation patterns on different genomic regions could influence the plant defence gene response and furthermore obtain distinct responses (Ali et al., 2020; Brocklehurst et al., 2018; Deleris et al., 2016; Dowen et al., 2012; Espinas et al., 2016; Hewezi et al., 2018; Köhler & Springer, 2017; López Sánchez et al., 2016; Ramos‐Cruz et al., 2021; Zhi & Chang, 2021). López Sánchez and authors reported that Arabidopsis hypomethylated Nuclear DNA‐dependent RNA polymerase V *nrpe1* (from the RdDM pathway) and hypermethylated Repressor of Silencing 1 *ros1* (DNA demethylation pathway) mutants presented a resistant and susceptible phenotype, respectively, when infected with *Hyaloperonospora arabidopsidis* and the presence or absence of DNA methylation could affect Salicylic Acid (SA)‐dependent defence against the pathogen (López Sánchez et al., 2016).

Chromatin organisation is another important epigenetic mechanism (Alonso et al., 2019; Berr et al., 2012; Panigrahi et al., 2021; Ramirez‐Prado, Piquerez, et al., 2018) and its role in plant–pathogen interactions was also highlighted recently. Several chromatin remodelling complexes have been analysed to play a role in the immune defence system against biotic stresses (Alonso et al., 2019; Panigrahi et al., 2021; Ramirez‐Prado, Piquerez, et al., 2018). SWItch/Sucrose Non‐Fermentable (SWI/SNF) and/or SWI2/SNF2‐Related 1 Chromatin Remodelling (SWR1) complex subunits are some of the chromatin remodelers that have important functions in the defence system, as well as a regulatory impact on SA and Jasmonic Acid/Ethylene (JA/ET) pathways (Berriri et al., 2016; March‐Díaz et al., 2008; Panigrahi et al., 2021; Ramirez‐Prado, Piquerez, et al., 2018). Also, histone modification enzymes have been identified as influencers of the defence related networks, such as JA/ET, Abscisic Acid (ABA) and/or SA‐dependent pathways, which, therefore, could contribute to a resistance or susceptible phenotypic behaviour after the biotic stresses (Chen et al., 2020; De‐La‐Peña et al., 2012; Ding & Wang, 2015; Kim, 2021; Ramirez‐Prado, Piquerez, et al., 2018).

Another important epigenetic mechanism is the RNA‐directed DNA methylation (RdDM) which acts parallelly with other epigenetic mechanisms for plant development and defence response (Ali et al., 2020; López et al., 2011; Zhu et al., 2016). It is known that RdDM is involved in the DNA methylation and RNAi pathways as a plant defence mechanism, specifically reported in plant–viruses interactions (Erdmann & Picard, 2020). López and colleagues have observed that Arabidopsis mutant for several different components of the RdDM pathway (especially RNA Pol V), when infected with bacteria or fungi, presented a resistant and susceptible phenotype, respectively, and opposite regulation of the SA and JA pathways (López et al., 2011). Weiberg and colleagues identified, in *Botrytis cinerea* Pers.: Fr., small RNAs (sRNA) that silenced the plants RNAi machinery, specifically Argonaute 1, to undermine and weaken the host's immune defence system (Weiberg et al., 2013). Interestingly, the same research group reported that in RNAi transgenic *B. cinerea‐Dicer‐like* 1/2 (*DCL 1/2*) gene in Arabidopsis and tomato plants (without host DCL change) presented a lower disease manifestation and fungus *DCL* gene expression. It indicated that the hosts could counterattack pathogens through the same RNAi strategy, which might be a possible novel agronomic approach through RNA‐directed fungicides (Wang et al., 2016).

Epigenetic machinery has also been analysed as playing a role in the plant pathogen's development, pathogenesis and metabolism (He, Zhang, et al., 2020; Zhu et al., 2016). Interestingly, Chen and colleagues reported that DNA methylation marks on *Phytophthora* oomycetes (positioned at the N6‐methyladenine [6 mA]) were influenced by their lifecycle and important virulent elements (effectors and transposable elements [TE]), suggesting that the activation of these patterns could lead to pathogen adaptation to plant defence (Chen et al., 2018; Rojas‐Rojas & Vega‐Arreguín, 2021). Therefore, the DNA methylation mechanism influences plants and pathogens from an adaptive response perspective by expanding their phenotypical and genomic range for either organism's survival.

In grapevine, little is known about epigenetic regulation in the context of its interaction with pathogens, namely with *P. viticola*. To further understand the epigenetic regulation mechanisms underneath grapevine–*P. viticola* interaction, a 5‐mC DNA methylation analysis was done during the first hours of contact between grapevine crossing hybrids and *P. viticola*. We have further looked into epigenetic and defence‐related transcripts modulation during this interaction in order to characterise the main mechanisms behind grapevine response to the downy mildew pathogen.

## 2 MATERIALS AND METHODS

### 2.1 Evaluation of susceptibility levels to *P. viticola* infection

We performed both a leaf disc assay and an *in planta* test using three *V. vinifera* cultivars (Italia, Red Globe and Crimson seedless) and four new genotypes of *V. vinifera*, derived from the cross between cultivar Red Globe (female) and Regal seedless (male) (N20/020, N23/018, N20/012 and N20/029). We chose these cultivar/new genotypes because, in previous field observations conducted at the Consiglio per la ricerca in agricoltura e l'analisi dell'economia agraria, Centro di Ricerca Viticoltura ed Enologia (CREA‐VE) (Turi, Italy), they showed different susceptibility levels to *P. viticola* infections on bunches. Bunches of 115 new genotypes from the cross Red Globe × Regal Seedless (5 plants/genotype) were observed starting from the phenological phases BBCH 61 (time of beginning of bloom) to the phenological phase BBCH 71 (time of beginning of berry ripening). For each genotype, the Disease Incidence (DI%), calculated as the percentage ratio between the number of bunches showing symptoms and the total number of bunches, and the Disease Severity (DS%) were calculated. In particular, the severity of downy mildew (was evaluated by using an empirical 0‐to‐5 rating scale (where 0 = bunch without symptoms, 1 = bunch with a damaged surface between 5 and 20%, 2 = bunch with a damaged surface between 25 and 40%, 3 = bunch with a damaged surface between 45 and 60%, 4 = bunch with a damaged surface between 65 and 80%, 5 = bunch with a damaged surface between 85% and 100%) and the formula of Townsend‐Heuberger (Lo Scalzo et al., 2012). Collected data were subjected to Cluster Analysis using the K‐means algorithm and the different genotypes were divided into three different clusters based on the level of tolerance/susceptibility to downy mildew infections (Figure S1). Genotypes N23/018, N20/012 and Red Globe showed low levels of both DI and DS, in contrast to the genotypes N20/020, N20/029 and Regal seedless that showed high levels of both disease indices (Table 1).

**TABLE 1:** Percentage of disease incidence (DI) and severity (DS) of *P. viticola* infection in table grape varieties and crossings

Table grapes		Grape bunch (field observations)	Leaf (disc assay)	Leaf (in planta assay)
		DI/DS (%)	DI/DS (%)	DI/DS (%)
Varieties	Regal Seedless	90.5/74.0 C1	100.0 a/93.6 a	–/–
	Italia	21.5/1.6 C2	71.4 ab/64.2 bc	47.5 ab/27.1 ab
	Crimson Seedless	6.7/1.1 C2	100.0 a/85.7 ab	61.9 ab/15.6 ab
	Red Globe	37.1/12.6 C2	61.9 ab/52.4 bc	56.8 b/21.6 b
Crossings	N20/012	34.0/16.6 C2	62.0 ab/25.2 c	58.3 ab/19.4 ab
	N20/020	79.3/46.5 C1	85.7 ab/80.3 ab	51.5 ab/19.4 ab
	N20/029	76.0/47.9 C1	100.0 a/79.6 abc	35.0 ab/10.7 ab
	N23/018	38.3/19.1 C2	35.7 b/27.6 c	22.1 a/5.3 a

*Note*: Data on grapevine varieties and crossings derived from the cross ♀red globe × ♂regal seedless are reported. The grape bunch analysis of the entire cross population and its statistical assessment is reported in the Figure S1. Genotypes belonging to C1 cluster showed higher susceptibility to *P. viticola*, compared to C2 (lower susceptibility), as assessed by cluster analysis. In the leaf disk assay, the four crossing genotypes represent a selection of 40 individuals analysed. The values followed by different letters are statistically different according to non‐parametric Conover's test.

#### 2.1.1 Leaf disc assay

**FIGURE 1:**
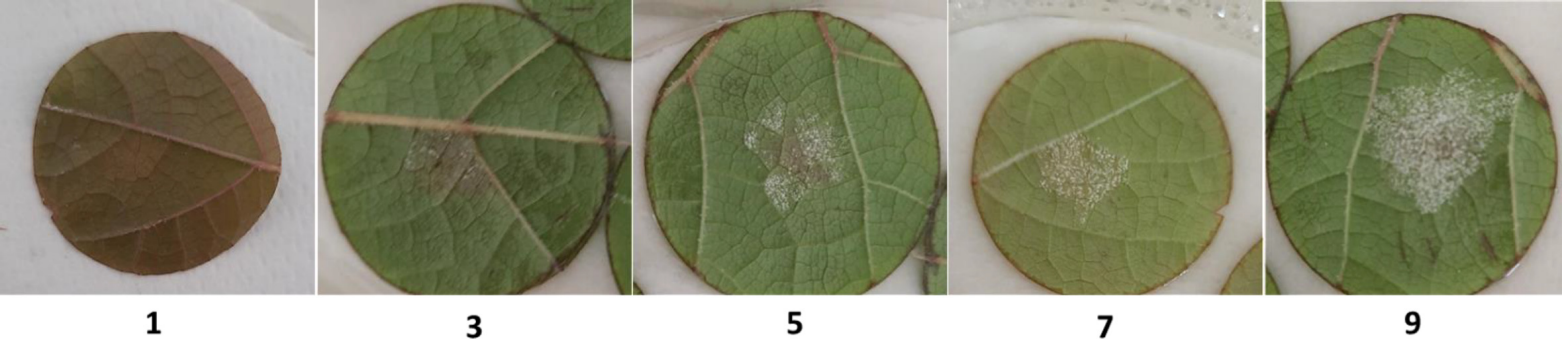
Rating scale applied for the phenotypic evaluation of *Plasmopara viticola* sporulation in leaf disc assays. 1: Absence of sporangiophores; 3: Sparse sporangiophores, localised in the inoculation point; 5: Patches of sparse sporulation intermixed with necrotic flecks underneath sporalting area; 7: Dense sporulation limited to inoculation point; 9: Dense sporulation beyond the point of inoculation

We performed a leaf disc assay, in order to confirm the contrasting susceptibility to *P. viticola* of selected individuals. In particular, the third, fourth and fifth fully expanded leaves beneath the apex were detached from each selected individual. From each leaf, 2.4 cm diameter discs were excised with a cork borer and placed randomly onto wet paper in Petri dishes with the abaxial side up. Three Petri dishes per individual, each containing seven leaf discs, were used. The *P. viticola* sporangia were collected from symptomatic grapevine leaves by brushing the white mould present on the underside of the leaves and each disc was inoculated with 50 μl of a sporangia suspension (1.8 × 10^4^ sporangia/ml). Sealed Petri dishes were incubated for 5 days at 20°C with a photoperiod of 8/16 h dark/light, in a culture chamber. Phenotypic observations were made on day 5. In particular, for each selected individual, the number of infected leaf discs were counted and, using a rating scale reported in Figure 1, the degree of sporulation was recorded for each leaf disc. Finally, for each individual, the Disease incidence (DI) (Ahmed, 2018) and disease severity (DS) were calculated by complying with the formula of Townsend‐Heuberger (Lo Scalzo et al., 2012).

#### 2.1.2 *In planta* assay

Wood cuttings from both selected cultivars (Italia, Red Globe and Crimson Seedless) and new genotypes (N20/020, N23/018, N20/012 and N20/029) were obtained from the experimental vineyard of the CREA‐VE, located in Rutigliano (Apulia region). Grapevine cuttings were harvested, cut in similar sizes (with 2 buds per cutting) and partially immersed in a 30°C water bath within a 4°C chamber for 2 months. This system allows the radicular system to grow without the rapid development of the rest of the plant. Then, the cuttings were transferred to square plastic pots with a mixture of seedling substrate and perlite (3:1 v/v). The wood cuttings were placed in a greenhouse for 2 months with natural light conditions at a temperature between 5°C and 30°C. The *P. viticola* sporangia were collected as previously described (paragraph 2.1.1), and sporangia suspension (1.8 × 10^4^ sporangia/ml) was sprayed on the abaxial side of the leaves (Figueiredo et al., 2017) of the different grapevine genotypes. As a control of the in vivo assay, plants were sprayed with water (mock inoculation). After inoculation, plants were covered with plastic bags to create a moist chamber with high humidity and kept in the dark for 8 h. Then, plants were maintained under greenhouse conditions. At 4, 6, 24 and 48 h post infection (hpi), the third to fifth fully expanded leaves were collected, frozen in liquid nitrogen and stored at −80°C. For each cultivar and time point (inoculated and mock inoculated), three biological replicates were collected consisting of three leaves from three different plants.

A modified disease development scale from OIV descriptor 452‐1 (leaf resistance degree) was used to evaluate the disease symptoms for the phenotypic analysis of the inoculated plants. The OIV descriptor scale was adjusted to six levels from 0 (no oil spots present) to 5 (85–100% of the surface of the leaf with oil spots) (OIV, 2009). Disease incidence (DI) (Ahmed, 2018) and disease severity (DS) were calculated, as described above, by complying with the formula of Townsend‐Heuberger (Lo Scalzo et al., 2012). Since data did not follow a normal distribution (Shapiro–Wilk's test) and homogeneity of variances (Levene's test), a non‐parametric statistic approach was used. In particular, permutational multivariate analysis of variance (PERMANOVA test [Anderson, 2017], one‐way ANOVA on ranks [Kruskal–Wallis *H* test (Kruskal & Wallis, 1952)]) and Conover's test of multiple comparisons using rank sums as post hoc test were used to assess the statistical differences between experimental conditions. All the statistical analyses, as well as a descriptive analysis of the data, were performed in R studio (version 3.5.0).

### 2.2 Evaluation of global 5 mC methylation % and transcriptome analysis in grapevine–*P* *. viticola* interaction

For the evaluation of the 5‐mC methylation plant profile and transcriptome analysis, leaf samples collected at 6 and 24 h from the inoculated and mock inoculated crossing genotypes N20/020 (S—susceptible) and N23/018 (T—tolerant) were used. These two genotypes were selected for molecular analyses, as in all the performed evaluations (field observation, leaf disc assay and *in planta* assay) they showed a constant phenotypic response to downy mildew infection (Table 1).

#### 2.2.1 DNA methylation assay

Genomic DNA isolation was performed using NucleoSpin Plant II (Macherey‐Nagel) according to the manufacturer's instructions. Genomic DNA quality was evaluated at A260/280 nm and A260/230 nm using a spectrophotometer (NanoDrop‐1000, Thermo Scientific). Prior to the global DNA methylation evaluation, the DNA of the biological replicates was combined with 0.5 μg of total DNA.

The 5‐mC DNA ELISA Kit (Zymo Research) was used to measure the percentage (%) of 5‐methylcytosine (5‐mC) present in the genomic DNA according to the manufacturer's instructions. An Anti‐5‐mC monoclonal antibody that is both sensitive and specific for 5‐mC was used. A horseradish peroxidase (HRP) conjugate was used as a secondary antibody. The absorbance was read at 405 nm after approximately 20 min of colour development. Each sample was assessed in triplicate. According to the manufacturer's instructions, the percent 5‐mC in a DNA sample was quantified from a standard curve generated with *Escherichia coli* DNA methylated with a CpG methylase. Considering that the density of CpG dinucleotides varies between species, in order to quantitate the percentage of 5‐mC, the calculated % 5‐mC was multiplied by the fold difference in CpG density between *E. coli* and the sample species. Therefore, the % CpG methylation of each sample was corrected by the CpG/genome length ratio of *V. vinifera* (4.4749). To determine the % of global methylation, the % CpG methylation was multiplied by the ratio of *V. vinifera* total cytosines/genome length (0.1483). The percentage of global 5‐mC methylation for each sample was calculated as fold change of inoculated *versus* mock (control). Statistical analysis was also performed to compare the fold change between N20/020 and N23/018 at each time‐point, using a Welch's *t* test from GraphPad Prism software version 8.2.1 (Pereira et al., 2022).

The contribution of *P. viticola* DNA methylation was not taken into account for the global methylation calculations, as in Pereira et al. (2022). It is reported that in oomycetes the methylation of adenines (6 mA) is the more frequent DNA methylation modification (Atighi et al., 2020; Chen et al., 2018; Pereira et al., 2022).

#### 2.2.2 Microarray analysis

Total RNA extraction was performed using the Agilent Plant RNA Isolation Mini kit (Agilent Technologies) according to the manufacturer's protocol. The total RNA purity and concentration were analysed by spectrophotometry (Nanodrop), 2100 Bioanalyzer (Agilent Technologies) and Qubit 4 Fluorometer (Invitrogen). A total of 1500 ng were used for cDNA synthesis. Both cDNA synthesis, labelling and hybridization procedures were made in accordance with the manufacturer's instructions (Agilent Technologies Protocol version 6.9.1).

A custom *V. vinifera* Agilent array with 44,000 probes (Catacchio et al., 2019) was used. The Agilent Feature Extraction software version 12.0 (Agilent Technologies) was used for data extraction. GeneSpring MultiOmics Analysis version 14.9 (Agilent Technologies) was used for the data analysis. Data was normalised through a percentile shift of 75%, baseline median of all samples and different types of filters (expression, flag, data file and error—coefficient of variance <20%). To improve the reproducibility of our microarray gene expression, a resampling in silico approach was performed on the outliers based on the workflow of the Sincell R work package as described by Juliá and colleagues (Juliá et al., 2015) applied to the microarray gProcessed Signals. To test the robustness of this strategy Pearson Correlation was performed.

For the statistical analysis, the Rank Product (RP) method was used to determine the total number of differentially expressed genes (DEGs) (Breitling et al., 2004) through a comparison between the RP of 1000 balanced permutations, the RP value of each gene and the comparison of Inoculation versus Mock conditions has a control subset. The DEGs cut‐off determination was based on the False Discovery Rate (FDR) < 0.17. In order to view the pathways that belong to the obtained DEGs, *V. vinifera* annotation was queried as described by Catacchio and colleagues (Catacchio et al., 2019) and complemented with V3 annotated *V. vinifera* version (https://urgi.versailles.inra.fr/Species/Vitis/Annotations) as well as NCBI Blast (https://blast.ncbi.nlm.nih.gov/Blast.cgi).

The Cytoscape platform v3.7.2 and ClueGo plug‐in v2.5.5 (Bindea et al., 2009; Shannon et al., 2013) were used for gene network analysis by using the V1 *V. vinifera* gene annotation. The Gene Ontology (GO) hierarchy was restricted between GO term levels 0 to 20. Kappa Score grouping was applied, as well as the Kappa Score Threshold of 0.4. To enrich the GO analysis, the AMIGO2 v2.5.13 platform (http://amigo.geneontology.org/amigo) was used. An overview of DEGs intersection between different experimental conditions was possible by the usage of Venn Diagram tools (http://bioinformatics.psb.ugent.be/webtools/Venn/). This multiple annotation method allowed to improve the identification of the epigenetic‐related DEGs (ER‐DEGs) in targeted pathways for further analysis. Subsequently, the data was reported as hierarchical clustering and heatmap of the defence‐related (DR‐) and ER‐ gene expression profile (gene microarray normalised intensities) through the Multiple Experiment Viewer (MeV) (Howe et al., 2010), applying the average linkage clustering and Pearson Correlation as the distance metric. Moreover, the pathways significantly modulated through ClueGo plug‐in v2.5.5 pathways (biological process, cellular component, molecular function and KEGG pathway) were identified with the fusion of GO terms based on gene similarities.

### 2.3 Quantitative real‐time PCR

Six ER‐DEGs were selected and their expression was validated by quantitative real‐time PCR (qPCR). Complementary DNA was synthesised from 1 μg of RNA using SuperScript IV Reverse Transcriptase kit (Invitrogen, Thermo Fisher Scientific) and anchored oligo(dT)16, according to the manufacturer's instructions. Expression analysis was performed with an ABI Prism 7900HT instrument (Applied Biosystems, Thermo Fisher Scientific) according to the manufacturer's protocol and using the Platinum SYBR Green qPCR SuperMix‐UDG with ROX (Invitrogen, Thermo Fisher Scientific) and gene‐specific primers (Table S1). All reactions were performed in triplicate. After each assay, a dissociation kinetics analysis was performed to verify the specificity of the amplification products. Relative amounts of all mRNAs were calculated using the 2^−ΔΔ*Ct*^ method (Livak & Schmittgen, 2001), where Δ*Ct* = *Ct* (target gene) − *Ct* (reference gene). The housekeeping gene actin was used as an endogenous reference for normalisation.

## 3 RESULTS

### 3.1 *Vitis vinifera* table grape crossing populations can portray susceptible or tolerant characteristics against *Plasmopara viticola* infection

A leaf disc assay and an artificial inoculation test on potted plants (*in planta* assay) were performed in order to confirm the different responses to *P. viticola* infection evaluated in preliminary studies. Results of all the experiments (Table 1 and Figure S1) reveal a different susceptibility between bunches and leaves to *P. viticola* infection for most of the genotypes analysed, as previously reported by other authors (Savary et al., 2009). The genotypes N20/020 and N23/018 were the most contrasting genotypes regarding disease incidence (both in leaves and bunches) and therefore selected for molecular studies. In the following experiments, these genotypes were referred as susceptible (S)—N20/020 and tolerant (T)—N23/018.

### 3.2 DNA methylation pattern differs between tolerant and susceptible genotypes

**FIGURE 2:**
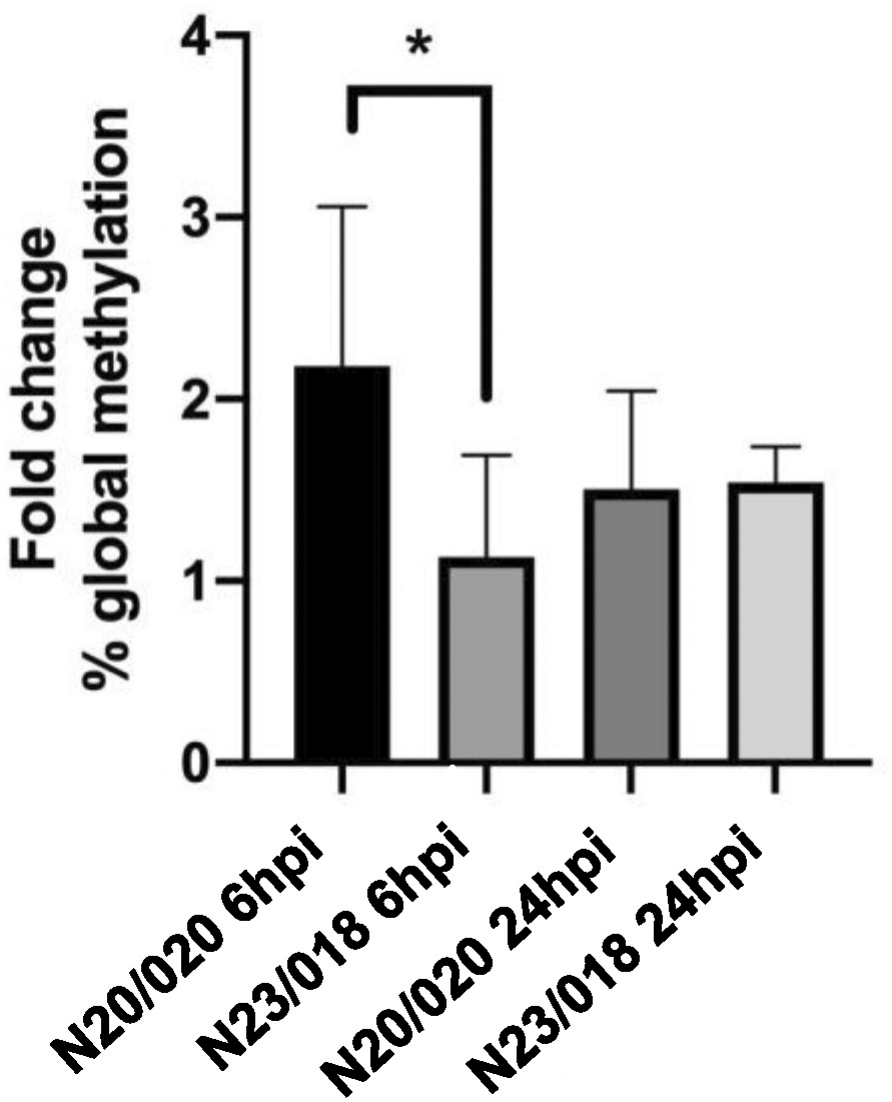
Fold change of the percentage of global 5‐methylcytosine (5‐mC) when comparing inoculated and mock conditions at 6 and 24 h post infection (hpi). Asterisk (*) represents the significant difference (*p* ≤ 0.05) between the susceptible (N20/020) and tolerant (N23/018) genotypes analysed

To assess if the global DNA methylation pattern reflects the contrasting susceptibility towards *P. viticola* of the two crossing hybrids, an evaluation of the percentage of the 5‐methylcytosine (5‐mC) was performed at 6 and 24 hpi. The global methylation pattern differs when comparing the susceptible (N20/020) and tolerant (N23/018) genotypes. The fold change reflects the comparison between the % 5‐mC levels in inoculated and mock inoculated samples. At both 6 and 24 hpi the % 5‐mC levels are higher in both genotypes (positive fold change) (Figure 2). However, the % 5‐mC alteration in the tolerant genotype was lower than in the susceptible genotype. The N20/020 presents higher modulation of the % 5‐mC levels at 6 hpi. At 24 hpi, modulation of the % 5‐mC is similar in both genotypes (Figure 2).

### 3.3 Transcriptome modulation at the first hours of interactions with *P. viticola*

**FIGURE 3:**
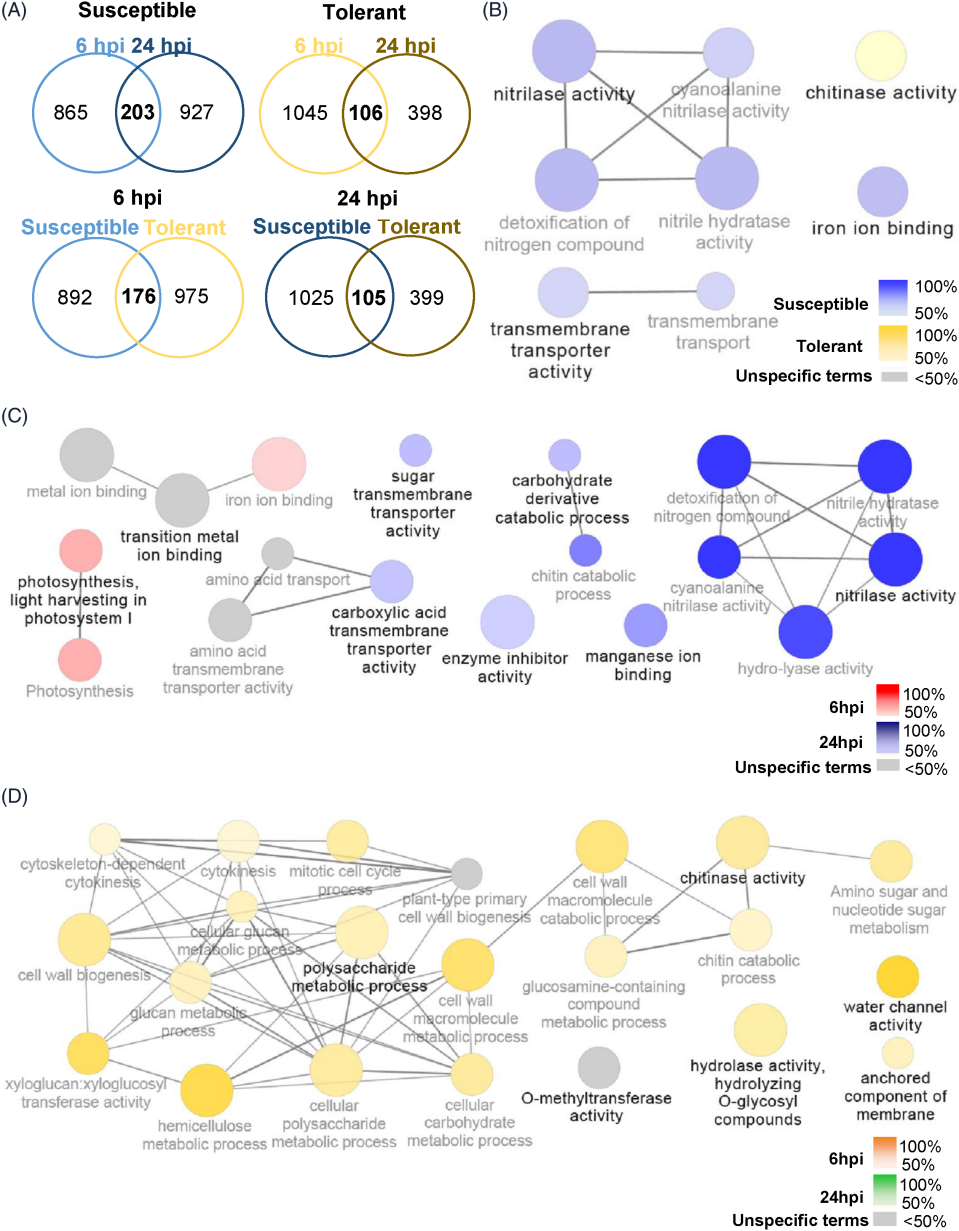
Identification of grapevine genes differentially expressed following *Plasmopara viticola* infection. Venn diagrams and network analysis of differentially expressed genes (DEGs) obtained from the comparison between inoculated and control (mock) samples. (A) Venn diagrams show differentially expressed genes at different hours post inoculation (hpi) in susceptible and tolerant genotypes. (B–D) Cluster distribution network of DEGs showing the GO terms significantly represented in N20/020 (susceptible) versus N23/018 (tolerant) genotypes (B) and those significantly represented in the comparison between 6 and 24 hpi in the susceptible (C) and the tolerant (D) cultivars. Gene network analysis was performed by Cytoscape plug‐in ClueGo. Only significant (*p* < 0.01) terms belonging to GO biological process, GO cellular components, GO molecular function and Kegg ontologies are shown. The colour gradient shows the gene proportion of each cluster with at least five genes on a GO interval from four to eight levels. Equal proportions of the two clusters are represented in grey. The node size is proportional to the term significance

Microarray assay was performed with RNA isolated from leaves of susceptible and tolerant genotypes, collected at 6 and 24 hpi upon inoculation with *P. viticola*. Mock inoculated samples were also analysed. To identify if the transcriptome modulation after *P. viticola* inoculation differs between N20/020 (S) and N23/018 (T), a cDNA microarray approach was followed. A custom *Vitis vinifera* microarray with 44,000 probes was used. Genes significantly modulated in the inoculated samples compared to mock were considered as differentially expressed (DEGs). Overall, 3108 genes were shown to be modulated (FDR <0.17), 1995 in the susceptible genotype and 1549 in the tolerant one (Figure 3A and Table S2). In N20/020, the number of DEGs is consistent between the time‐points analysed (1068 at 6 hpi and 1130 at 24 hpi), in N23/018, transcriptome modulation is higher at 6 hpi, with the majority of the DEGs being down‐regulated (Table 2).

**TABLE 2:** Overview of the differentially expressed genes (DEGs) after *P. viticola* infection of the susceptible (S—N20/020) and tolerant (T—N23/018) genotypes

Conditions	Total DEGs	Up‐regulated	Down‐regulated
S6hpi	1068	611	457
S24hpi	1130	839	291
T6hpi	1151	371	782
T24hpi	504	239	265

*Note*: Up‐ or down‐ regulation of genes was observed through the comparison of inoculated versus mock conditions.

When comparing both genotypes, at 6 and 24 hpi in both time points, the majority of the DEGs are specific to each interaction. At 6 hpi only 176 DEGs are commonly modulated and at 24 hpi 105 DEGs are commonly modulated (Figure 3A). Within the genes commonly modulated at 6 hpi, 37 are related to plant defence responses (DR‐DEGs). Meanwhile, at 24 hpi, 21 DEGs belong to the defence related data set. The DR‐DEGs were characterised as GO terms related to defence and stress response, hormone biosynthesis and signalling categories.

To increase the knowledge of the functional networks affecting grapevine response to *P. viticola*, between N20/020 and N23/018, a gene network analysis was conducted on Cytoscape plug‐in ClueGo (Figure 3B). A total of 33 clusters were significantly enriched (*p* < 0.01), with the N20/020 genotype presenting a significantly higher percentage of DEGs involved in nitrilase activity, iron ion binding activity and transmembrane transporter activity, when compared with the tolerant genotype. The N23/018 genotype showed a significant modulation of genes involved in chitinase activity (Figure 3B).

The networking analysis of DEGs, at each time point, showed that different biological pathways and molecular functions are affected during the time course (Figure 3C,D). For instance, in the susceptible variety at 6 hpi, a higher number of DEGs are associated with iron ion binding processes and photosynthesis processes (Figure 3C). Several processes connecting to manganese ion binding, enzyme inhibitor activity, carbohydrate derivative catabolic process, sugar transmembrane transporter activity, carboxylic acid transmembrane transporter activity and nitrilase activity are also enriched in the susceptible genotype (Figure 3C). Regarding the tolerant genotype, polysaccharide metabolic processes, anchored components of membrane, chitinase activity, water channel activity, hydrolase activity, hydrolyzing O‐glycosyl compounds are GO terms significantly modulated at 6 hpi (Figure 3D). Six DEGs were analysed by qPCR in order to validate microarray data (Figure S2). The analyses confirmed the expression trend observed.

**FIGURE 4:**
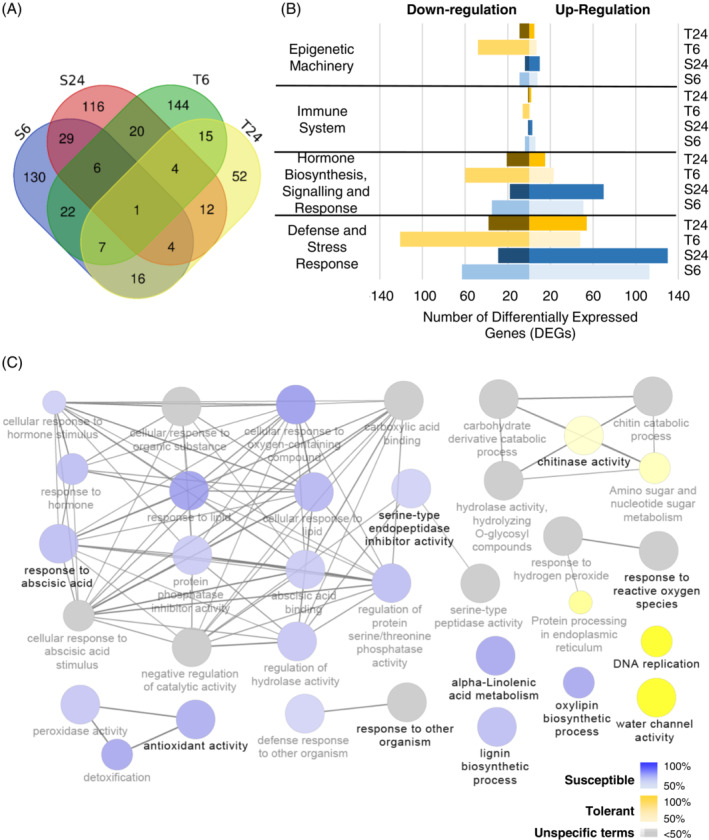
Defence response modulation after *Plasmopara viticola* inoculation. (A) Venn diagram showing the distribution of defence related differentially expressed genes (DR‐DEGs) at 6 and 24 h post inoculation (hpi) in susceptible (S—N20/020) and tolerant (T—N23/018) genotypes. Genes significantly modulated in the inoculated versus mock were considered as differentially expressed for each sample. (B) Histogram of the DR‐DEGs grouped by the most representative GO terms related to plant defence responses. Up‐ and Down‐regulation of inoculated versus mock is attributed by the rank product statistical method. T24: Tolerant at 24 hpi; T6: Tolerant at 6 hpi, S24: Susceptible at 24 hpi; S6: Susceptible at 6 hpi. (C) Cluster distribution network of DR‐DEGs showing most represented defence pathways when comparing susceptible (blue) versus tolerant genotypes (yellow). Gene network analysis was performed by Cytoscape plug‐in ClueGo. Only significant (*p* < 0.01) terms belonging to GO biological process, GO cellular components, GO molecular function and Kegg ontologies were shown. Gene proportion of each cluster is presented by colour gradient containing at least five genes on a GO interval from four to eight levels. Equal proportions of the two clusters are represented in grey. The node size is proportional to the term significance

It is well established that *P. viticola* inoculation leads to a broad modulation of grapevine transcriptome (Figueiredo et al., 2012; Polesani et al., 2010). In our study, a total of 578 genes were identified as being associated with grapevine defence (DR‐DEGs), corresponding to 18.4% and 19.6% of the total DEGs for susceptible and tolerant genotypes, respectively (Figure 4A and Table S3). In N20/020, genes related to defence and DR signalling pathways were mainly up‐regulated at both time points. In this genotype, the number of signalling related genes was higher after 24 h of the pathogen inoculation (Figure 4B). Meanwhile, in the tolerant genotype, a predominant down‐regulation of the defence genes was observed at 6 hpi and signalling related genes were mainly down‐regulated at both time points (Figure 4B).

To assess if the level of plant susceptibility/tolerance to this pathogen is related to different defence mechanisms, a gene network analysis of DR‐DEGs was conducted (Figure 4C). Although numerous GO terms were equally represented in the two genotypes (grey clusters), a number of biological processes and molecular functions were more represented in S or T genotypes. For instance, genes involved in DNA replication and chitinase activity were more represented in T DEGs (Figure 4C). Genes related to signalling pathways were found to be differentially modulated in our study after pathogen colonisation. The susceptible genotype, at 24 hpi, presents more signalling related genes with a high fold change than at 6 hpi. Meanwhile, the tolerant variety has more DEGs related to signalling pathways at 6hpi (Table S4). Metabolism plays a role in different levels of defence responses. In this study, we found that genes involved in both primary and secondary metabolism were modulated in grapevine–*P. viticola* interaction. Most of them were up‐regulated in the susceptible genotype at 24 hpi (Table S4), suggesting that the infection has less impact on the metabolic related genes of the tolerant genotype.

Both genotypes showed significant differential expression of genes encoding for important classes of genes involved in plant defence against pathogens, such as pattern recognition receptors (PRR‐like), pathogenesis‐related (PR) proteins and disease resistance proteins (Table S4 and Figure S3). The susceptible variety showed that ribonuclease‐like PR10 and lipid transfer PR14 genes were differentially modulated. Meanwhile, the tolerant genotype presented several different pathogenesis‐related genes significantly down‐regulated early after pathogen colonisation. These data show that the PR proteins were mostly down‐regulated in the two genotypes studied. Nevertheless, there was a higher modulation of genes encoding PR proteins on the tolerant variety (Table S4).

### 3.4 Grapevine epigenetic‐related (ER) genes were differentially modulated in N20/020 and N23/018

**FIGURE 5:**
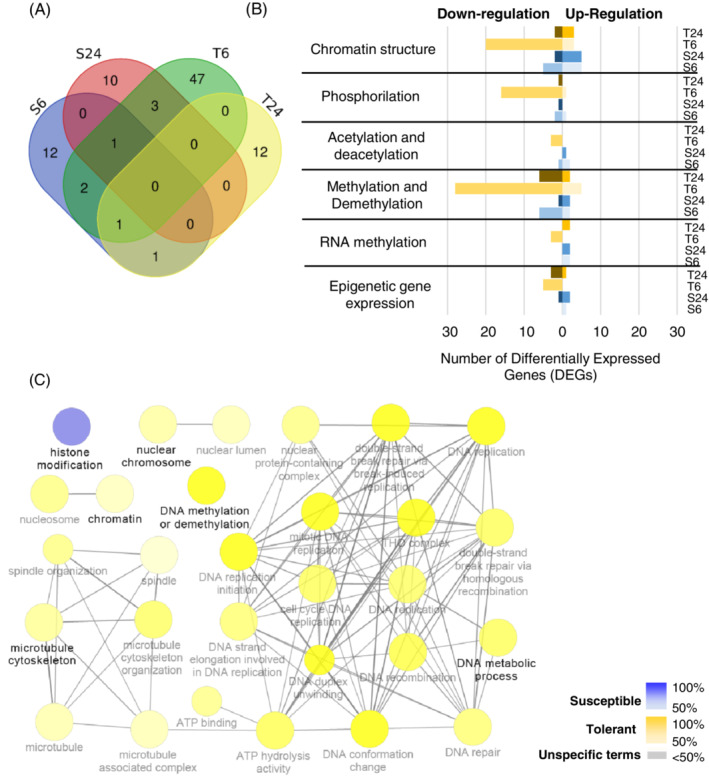
Modulation of epigenetic related genes following *Plasmopara viticola* infection. (A) Venn diagram showing the number of epigenetic related differentially expressed genes (ER‐DEGs) at 6 and 24 h post inoculation (hpi) in susceptible (S—N20/020) and tolerant (T—N23/018) genotypes. Genes significantly modulated in the inoculated versus mock were considered as differentially expressed for each sample. (B) Histogram of the ER‐DEGs grouped by the most representative GO terms related to epigenetic machinery. Up‐ and Down‐regulation of inoculated versus mock is attributed by the rank product statistical method. T24: Tolerant at 24 hpi; T6: Tolerant at 6 hpi, S24: Susceptible at 24 hpi; S6: Susceptible at 6 hpi. The following processes were considered for each GO subcategory. Chromatin structure: Organisation, modification, regulation, involvement and histone modification; Phosphorilation: Histone biological process and post‐translational modifications; acetylation and deacetylation: Histone post‐translational modifications, pathways, transferase complexes and activity; methylation and demethylation: DNA and histone pathways, maintenance, regulation, enzyme activity and modifications; RNA methylation: Pathways, complexes and enzyme activity; epigenetic gene expression: Regulation. (C) Network cluster distribution of ER‐DEGs showing the most significant pathways affected in grapevines after infection. Gene network analysis was performed by Cytoscape plug‐in ClueGo. Only significant (*p* < 0.01) terms belonging to GO biological process, GO cellular components, GO molecular function and Kegg ontologies were shown. Gene proportion of each cluster was presented by colour gradient containing at least four genes and of total genes per term as well as a GO interval from six to eight levels. Equal proportions of the two clusters are represented in grey. The node size is proportional to the term significance

Eighty‐nine genes differentially expressed in the inoculated samples, compared to mock, were identified as being associated with epigenetic regulation (ER‐DEGs), corresponding to 2% (susceptible) and 4% (tolerant) of the total DEGs identified (Figure 5A and Table S5). Among them, 25 genes were also classified as defence‐related (Table S6).

The susceptible genotype, at 6 h after inoculation, presented 17 modulated transcripts with GO annotation related to epigenetic machinery, while, at 24 hpi, the number decreased slightly (14) (Figure 5A). In contrast, in the tolerant genotype, a higher number of differentially expressed genes (54 ER‐DEGs) were observed at an early time point (6 hpi) (Figure 5A). The main GO terms associated with ER mechanisms after *P. viticola* inoculation were: methylation and demethylation and chromatin remodelling (Figure 5B). Considering the functional networks more represented in each genotype at different time points, genes related to histone modifications were significantly modulated in the susceptible genotype (Figure 5C), whereas, DNA methylation machinery, nuclear chromosome, chromatin microtubule cytoskeleton and DNA metabolic processes were significantly affected in the tolerant genotype (Figure 5C).

DNA methylation related genes such as *DNA Methyltransferase 1* (*MET1*), *Chromomethylase 1* (*CMT1*) and *Chromomethylase3* (*CMT3*) were all significantly down regulated in the tolerant genotype (Table 3). Genes involved in DNA replication, damage response and repair mechanisms are significantly modulated in this work (Table 3). Chromatin remodelers play an important role since they crosstalk with several different defence and developmental networks. Genes related to chromatin structure (as *ATPase SPLAYED*—*SYD, High Expression of Osmotically Responsive Genes 15*—*HOS15, Histone H2A 12*—*HTA12, HTA12.1, Histone H2A 8—HTA8 and linker Histone 1—H1*), histone methylation (e.g., *SU[var]3–9 homologue 5*—*SUVH5*) and acetylation (*Histone acetyltransferase of the GNAT family 2—HAG2.1, HAG2.2*) were differentially expressed after pathogen infection. Moreover, differences in their regulation were found between the two genotypes analysed. Epigenetic mechanisms besides chromatin, histone and DNA modifications are also influenced by RNA such as small RNAs. In this study, small RNAs coding genes such as *Arginine/Serine‐Rich Splicing Factor 40/41* (*RSP40/RS41*) and *DCL2* were differentially expressed after pathogen infection and also showed a different regulation in each genotype analysed (Table 3). Finally, genes involved in the DNA damage repair machinery (as *Retinoblastoma‐Related 1—RBR1, Gamma‐Histone variant H2AX—GAMMA‐H2A.X, RAD51*) were differentially expressed after pathogen inoculation.

**TABLE 3:** Epigenetic related genes (ER‐DEGs) differentially expressed after *P. viticola* infection in both N20/020 (S) and N23/018 (T) at 6 and 24 hpi

Gene ID	S6	S24	T6	T24	Gene name	Product	Gen Bank Acc.
*DNA damage/repair response*							
VIT_04s0008g02780		2.48			*RBR1*	PREDICTED*: Vitis vinifera* retinoblastoma‐related protein‐like (RBR)	XM_010650145.2
VIT_07s0104g00960			−8.07		Gamma‐*H2AX*	histone H2AX	XM_002271470.3
VIT_11s0016g00340			−2.40		*RAD51*	DNA repair protein RAD51 homologue	XM_002273767.2
*DNA methylation/demethylation*							
VIT_08s0007g06800			−2.39		*CMT1*	PREDICTED*: Vitis vinifera* putative DNA (cytosine‐5)‐methyltransferase CMT1	XM_002275896.3
VIT_06s0004g01080				−3.62	*CMT3*	DNA (cytosine‐5)‐methyltransferase CMT3	XM_010653042.1
VIT_12s0035g01770				−4.37	*MET1*	PREDICTED: *Vitis vinifera* DNA (cytosine−5)‐methyltransferase 1B‐like	XM_019223723.1
VIT_06s0061g01270				−5.27	*DME*	PREDICTED: *Vitis vinifera* transcriptional activator DEMETER	XM_019220413.1
*Chromatin remodelling*							
VIT_05s0020g02000				−4.47	*SYD*	PREDICTED*: Vitis vinifera* chromatin structure‐remodelling complex protein SYD	XM_010651496.2
VIT_11s0016g05490			−3.01		*ARP6*	Predicted: *Vitis vinifera* Actin‐related protein 6	XM_010658136.2
*Histone modification*							
VIT_11s0016g02620			−3.71		*HAG2.1*	histone acetyltransferase type B catalytic subunit	XM_002282895.2
VIT_13s0047g00150	2.03				*HAG2.2*	PREDICTED: *Vitis vinifera* histone acetyltransferase type B catalytic subunit	XM_002282895.3
VIT_00s0179g00340			−2.33		*HTA8*	PREDICTED: *Vitis vinifera* histone H2A variant 1	XM_002281230.2
VIT_16s0013g00310	−2.59				*SUVH5*	PREDICTED: *Vitis vinifera* histone‐lysine N‐methyltransferase, H3 lysine‐9 specific SUVH5	XM_002277738.3
VIT_18s0001g09610	−2.84				*HOS15*	PREDICTED: *Vitis vinifera* F‐box‐like/WD repeat‐containing protein TBL1Y	XR_002032367.1
VIT_06s0004g04270			−2.10		*HTA12*	histone H2A	XM_002284269.3
VIT_14s0060g02360		−10.82			*HTA12.1*	histone H2A.1	XM_002283935.3
VIT_07s0005g01060			−2.68		*H1*	histone H1	XM_002269443.3
*Small RNA biogenesis and RNA regulation*							
VIT_04s0023g00920				−4.10	*DCL2*	PREDICTED: *Vitis vinifera* endoribonuclease Dicer homologue 2	XM_019219502.1
VIT_15s0048g01870		2.66			*RSP40*	serine/arginine‐rich splicing factor RS41‐like	XM_002273715.3
VIT_11s0016g03220				2.58	*RDR5*	probable RNA‐dependent RNA polymerase 5	XM_010657967.1

**FIGURE 6:**
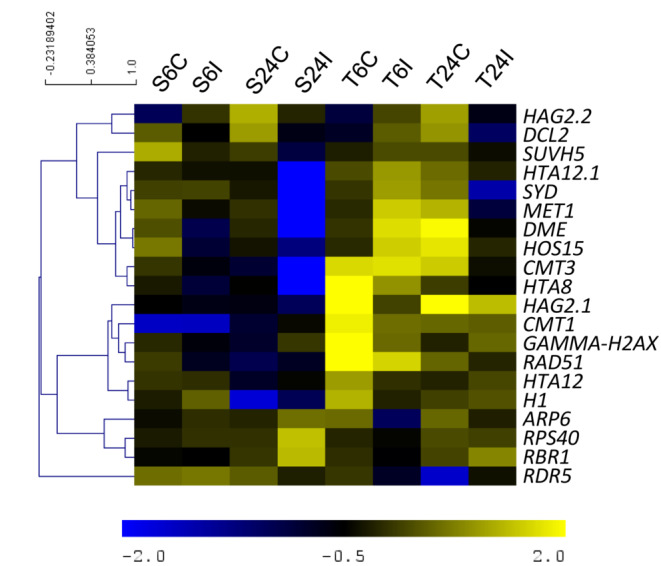
Hierarchical clustering of selected epigenetic related genes differentially expressed in grapevine leaves after *Plasmopara viticola* infection. Microarray expression data of the putatively susceptible (S) and tolerant (T) genotypes at 6 and 24 h post inoculation with different treatments, mock (C) and inoculated (I), were used for clustering analysis. A selection of ER‐DEGs significantly differentially expressed in the inoculated versus mock conditions is reported. For each gene, the Log_2_ normalised expression value was used. The different colours indicate different expression values, as reported in the colour bar. Lowest and highest expression values are in blue and yellow, respectively. Gene IDs of the selected genes are those reported in Table 3

The microarray expression profile/hierarchical clustering of the main ER‐DEGs was reported in Figure 6. It was possible to observe the formation of three main clusters, the first and most notable cluster includes the main epigenetic machinery such as *MET1, CMT3, DME, SYD* and *DCL2*.

## 4 DISCUSSION

The cultivated grapevine is highly susceptible to the downy mildew disease, which harbours heavy production losses. Several breeding programs are being conducted in order to introgress resistance traits into grapevine cultivars with economic interest (Boso et al., 2014; Boso & Kassemeyer, 2008; Marsico et al., 2018). In this study, we performed a more in‐depth assessment of our previous field observations regarding *V. vinifera*–*P. viticola* interaction. We evaluated the disease incidence and severity of *P. viticola* in three grapevine varieties and four crossing hybrids selected from the cross between Red Globe x Regal seedless for their high‐ or low‐susceptibility to the pathogen. Of the four crossing hybrids, N23/018 (T) was the most tolerant to *P. viticola* presenting lower DI and DS both in vitro and in vivo inoculations. The crossing hybrid N20/020 (S) was highly susceptible.

To further understand if epigenetic regulation is one of the key factors influencing the higher tolerance/susceptibility of the crossing hybrids, we have conducted a 5‐mC % evaluation in the first hours after pathogen contact. We have also performed a characterisation of transcriptome modulation focusing on both defence‐related (DR‐DEGs) and epigenetics related transcripts (ER‐DEGs).

The transcriptomic analysis of S and T was performed at 6 and 24 hpi, as pathogen recognition and development were described to occur in a short period of time after inoculation (Unger et al., 2007). A higher number of differentially expressed genes at 6 hpi was observed in the T genotype, resembling the timing and response of a resistant species to *P. viticola* inoculation (Figueiredo et al., 2012). Also, the low overlapping DEGs between S and T indicate a genotype‐specific response to pathogen stimulus. This is in accordance with the grapevine response to other kinds of stresses (Catacchio et al., 2019).

### 4.1 Modulation of defence pathways after pathogen infection

The recognition of pathogen molecules on the host cells is done through pattern recognition receptors (PRR) localised in plasma membranes that further initiate the first steps of immunity (Iqbal et al., 2021; Ramirez‐Prado, Abulfaraj, et al., 2018). By recognising pathogen‐associated molecular pattern (PAMP) molecules, they activate down‐stream signalling pathways responsible for the activation of plant defence mechanisms, a process called PAMP‐triggered immunity (PTI) (Parker et al., 2021; Ramirez‐Prado, Abulfaraj, et al., 2018). As observed in our work, PRR‐like genes, as well as genes involved in the down‐stream signalling pathway like mitogen‐activated protein kinase, were differentially expressed after pathogen inoculation in both genotypes. Differences in the modulation of these genes were observed between susceptible and tolerant genotypes, in accordance with their different phenotypic response.

Jasmonic acid (JA), abscisic acid (ABA) and ethylene (ET) pathways are important phytohormones with cascade networks which regulate the defence system of *V. vinifera* (Chong et al., 2008; Figueiredo et al., 2018). Genes encoding the signalling phytohormones that are involved in plant defence were overrepresented in our datasets, in accordance with their pivotal role in the modulation of plant defence responses (Chong et al., 2008; Figueiredo et al., 2018; Laureano et al., 2018). Signalling pathways, such as ABA, are considered host species dependent (Lievens et al., 2017). On the other hand, SA and JA/ET have a key role in the activation of plant defence response dependent on the pathogens feeding relationship with the host (He, Yuan, et al., 2020). Our results indicated that genes related to these pathways were modulated in the presence of the oomycete. However, differences between susceptible and tolerant varieties were also observed.

Transcriptional and post‐transcriptional modifications play an important role in the regulation of the activation of grapevine defence networks (Figueiredo et al., 2008). For instance, we found that genes encoding for transcription factors and those involved in protein modifications were differentially expressed after pathogen infection. Moreover, a modulation in the expression of important classes of genes involved in plant immunity, such as PTI‐ and ETI‐related genes, PR and disease resistance proteins were found after the pathogen inoculation, and differences between the two genotypes were observed in accordance with their different susceptibility level to *P. viticola*.

**FIGURE 7:**
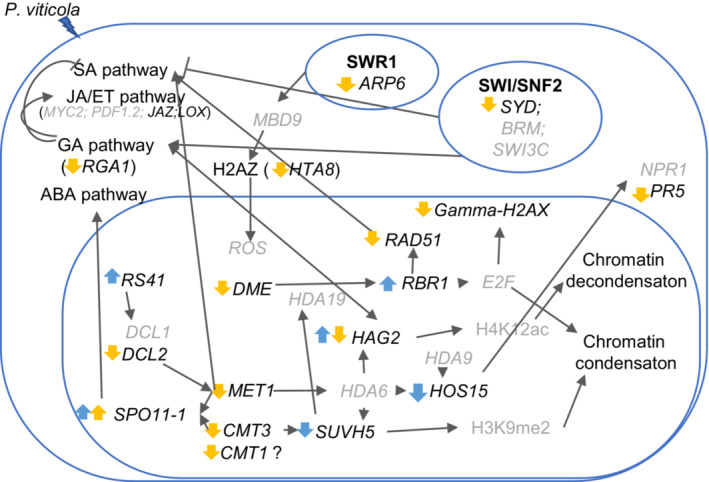
Schematic representation of the epigenetic machinery interaction and modulation after *Plasmopara viticola* infection of grapevine leaf tissue. Yellow arrows: Genes differentially expressed in the tolerant genotype. Blue arrows: Genes differentially expressed in the susceptible genotype. Up arrows indicate up‐regulation, down arrows indicate down‐regulation

Together with analysing the genes involved in defence responses, in this work, we focused on the DEGs involved in the plant epigenetic machinery and its interplay with its defence system (Figure 7). Evidence of the involvement of epigenetic modulation in plants resistance has been increasingly reported for a wide variety of biotic stresses (Cui et al., 2021; Ding & Wang, 2015; Elhamamsy, 2016; Espinas et al., 2016; Kong et al., 2020; Kuźnicki et al., 2019; Ramirez‐Prado, Abulfaraj, et al., 2018; Zhi & Chang, 2021).

### 4.2 DNA methylation plays a role in the plant immune defence system

In the stress adaptation mechanism, epigenetic modulation may play different roles. Potentially epigenetic mechanisms may either directly regulate defence networks or affect genome reorganisation and stability (Boyko & Kovalchuk, 2008). DNA methylation regulates gene expression in plant defence responses. Based on our results, we hypothesise that *V. vinifera*–*P. viticola* interaction influences epigenetic mechanisms such as DNA methylation maintenance and associated components such as DCL2. We observed a down‐regulation of genes encoding the DNA methyltransferases in T at 6 hpi (*CMT1*) and 24 hpi (*MET1* and *CMT3*). Reports on the lacking of either MET1 or CMT3 methyltransferase activity in genetic mutants of different plant species induce resistance after the interaction with an array of pathogens (viruses, fungus and bacteria) (Chen et al., 2018; Cui et al., 2021; Dowen et al., 2012; Geng et al., 2019; Kuźnicki et al., 2019; López Sánchez et al., 2016; Pujara et al., 2021). Also, DNA methylation can affect either SA pathway genes or *pathogenesis responsive gene 1 (PR1)* expression (Chan & Zimmerli, 2019; Dowen et al., 2012; Hewezi et al., 2018). Moreover, global methylation patterns also differ between the susceptible and tolerant crossing hybrids. In accordance with that, global methylation presented a lower increase in the tolerant genotype. Analysis of global DNA methylation pattern modification on several species after infection with different pathogens has been reported on tomato roots, Poplar bark and Arabidopsis leaves (Leonetti & Molinari, 2020; Xiao et al., 2021). Often, resistant species showed DNA hypomethylation patterns while susceptible ones presented hypermethylated DNA after pathogens attack (Leonetti & Molinari, 2020; Xiao et al., 2021). Also, studies have observed that altering DNA methylation levels (hypo‐ or hypermethylation) on genomic regions could influence gene and phenotypic responses to plant diseases (Geng et al., 2019; Leonetti & Molinari, 2020). Our results are in agreement with recent work done by Pereira and authors, which reported that the global cytosine analysis performed on the tolerant cultivar Regent revealed a decrease of global methylation levels in the early hours of infection by *P. viticola* in comparison to the Trincadeira, a susceptible genotype (Pereira et al., 2022).

### 4.3 Involvement of epigenetic mechanisms in grapevine defence response

Histone modifications are affected by the *P. viticola* inoculations early in the colonisation leading to a chromatin modification. This can, depending on the role of the histones, effect gene silencing or activation. A very important role in the epigenetic‐defence machinery is played by the chromatin remodelers (Alonso et al., 2019; Ding & Wang, 2015; Espinas et al., 2016; Ramirez‐Prado, Piquerez, et al., 2018; Song et al., 2021; Wang et al., 2020; Zhi & Chang, 2021). In our study, variation in the expression of chromatin remodelers subunits, such as SYD (part of the SWI/SNF chromatin complexes), could lead to alteration of chromatin conformation. Studies in *Arabidopsis thaliana* have shown that SYD is recruited to the promoter of a set of JA and ET responsive defence genes, and it is essential for their expression. Besides chromatin and DNA methylation, histone modifications are also involved in the plant defence responses (Ramirez‐Prado, Piquerez, et al., 2018). In our study, several genes related to histones modifications, encoding for structural or regulatory proteins, were differentially expressed after *P. viticola* infection (Table 3). An important gene encoding protein of the WD‐40 repeat family (*HOS15*) was downregulated in both analysed genotypes at different time periods of infection. HOS15 plays an important role in plant immunity against different stresses via histone acetylation/deacetylation pathways (Kumar et al., 2021). Li and colleagues observed, in wheat infected with powdery mildew, a negative regulatory role of HOS15 in the defence system (Liu et al., 2019). The authors suggested that a mediation between HOS15‐HDA6 and histone acetyltransferases could be occurring to regulate the transcription of defence related genes (Liu et al., 2019).

Furthermore, the *linker histone 1 (H1)* was downregulated at T genotype at 6 hpi. Histone H1 is known to regulate chromatin condensation (Sheikh et al., 2022). Sheikh and colleagues observed the effect of the absence of H1 in Arabidopsis when inoculated with *Pseudomonas syringae* and treated with flagellin flg22 for priming analysis (Sheikh et al., 2022). They eluded that the genes related to defence are influenced by H1 to respond immediately or not to the pathogen attack (Sheikh et al., 2022). Also, while analysing the role of DNA methylation and histone modifications within the plant immune defence system, Arabidopsis H1 indicated a possible role in plant immunity since it impacts chromatin rearrangement through the influence of the epigenetic profile (Sheikh et al., 2022). H1 is part of the defence and epigenetic machinery in our work.

Beyond the more studied epigenetic traits, RNA modification can influence plant defence responses and plant adaptation mechanisms. It is well known that Dicer‐like proteins are essential components of the miRNA and siRNA biogenesis and that these molecules have a recognised role in plant defence mechanisms (Qin et al., 2017). In our study, the repression of the *Dicer‐like 2 (DCL2)* gene in the T variety could suggest a role in grapevine–oomycete interaction. Studies have shown that DCL2 and DCL4 are associated with virus infection immune defence with the purpose of attaining viral siRNA (vsiRNA) as a strategy against viruses (Ashapkin et al., 2020; Erdmann & Picard, 2020; Lee & Carroll, 2018; Prakash et al., 2017). Also, Wang et al. analysed *B. cineria* interaction with a wide variety of hosts where it was identified a bi‐directional defence process between host and pathogen RNAi and *DCL2* targeted genes (Wang et al., 2016). Another important small RNA biogenesis molecule evident in our study is the *serine/arginine‐rich splicing factor RS41‐like (RSP40)* that were up‐regulated in the susceptible cultivar. Literature has shown that DCL1 plays a defence role in plant–*B. cinerea* interaction, whereas Coolen and colleagues observed that the expression of *DCL1* in RS40/41 mutant arabidopsis after *B. cinerea* infection decreased (Coolen et al., 2019; Weiberg et al., 2013). Retinoblastoma Related 1 (RBR1), RAD51 and gamma‐H2AX are related to the epigenetic machinery as well as interact with defence mechanisms against biotic stresses (Bouyer et al., 2018; Camborde et al., 2019; Desvoyes & Gutierrez, 2020; Wang et al., 2010, 2014). RBR1 has been revealed as a member of the DNA Damage repair machinery (DDR) together with RAD51 (Bouyer et al., 2018; Camborde et al., 2019). Furthermore, gamma‐H2AX and RAD51 affect the DNA through direct DSB when interacting with other plant‐necrotrophic pathogens (Song & Bent, 2014). Overall, these data support the involvement of these epigenetic mechanisms in the regulation of the grapevine defence response against *P. viticola* and open the possibility of new functional regulations of stress responses in grapevine.

## 5 CONCLUSIONS

So far, an in‐depth analysis of the response of table grape genotypes with different susceptibilities against *P. viticola* has been reported for the first time. The transcriptomic characterisation allowed us to observe the modulation of important classes of defence genes during *V. vinifera*–*P. viticola* interaction, as those encoding for proteins involved in PAMP perception, phytohormone signalling and response, PR proteins and defensin‐like proteins.

In this study, an observation of the epigenetic associated machinery was also performed. Our work indicates that the DNA methylation is affected by *P. viticola* inoculation and suggests that differences in the DNA methylation levels might be related to the different susceptibility to *P. viticola*. Moreover, a differential regulation of genes involved in chromatin and histone modification, small RNA biogenesis and DNA damage and reparation processes were also observed, suggesting that these processes may also play a role in the grapevine defence responses to this pathogen.

Finally, we found that the tolerant *V. vinifera* genotype analysed for the early modulation of defence genes and the effect on global DNA methylation resemble what has been observed in the resistant grapevine genotypes derived from other *Vitis* species, showing an intermediate behaviour between resistance and susceptibility. These data support the presence of tolerance mechanisms in *V. vinifera* and the presence of similar strategies to counteract *P. viticola* among different *Vitis* species.

## AUTHOR CONTRIBUTIONS

Vanessa Azevedo, Andreia Figueiredo and Fiammetta Alagna designed the experimental plan and wrote the manuscript with inputs from all the authors; Vanessa Azevedo, Loretta Daddiego and Lisete Sousa analysed the transcriptomic data; Vanessa Azevedo, Maria Francesca Cardone and Carlo Bergamini performed the microarray assays; Loretta Daddiego and Giorgio Perrella carried out the qPCR; Antonio Domenico Marsico and Fiammetta Alagna performed the leaf disc assays, Vanessa Azevedo, Antonio Domenico Marsico and Carlo Bergamini executed the in vivo assay and the phenotypic analysis; Rita B. Santos, Vanessa Azevedo, Andreia Figueiredo performed and analysed the DNA methylation assay; All the authors reviewed the manuscript.

## Supporting information


**Appendix S1**. Supporting information.

**Table S2**. Data set of defense related differentially expressed genes (DR‐DEGs). Genes modulated at 6 and 24 hpi in grapevine tolerant (T) and susceptible (S) genotypes are reported.

**Table S3**. Data set of defense related differentially expressed genes (DR‐DEGs). Genes modulated at 6 and 24 hpi in grapevine tolerant (T) and susceptible (S) genotypes are reported.

**Table S5**. Data set of epigenetic related differentially expressed genes (ER‐DEGs). Genes modulated at 6 and 24 hpi in grapevine tolerant (T) and susceptible (S) genotypes are reported.

## Data Availability

The data that support the findings of this study are openly available in Gene Expression Omnibus repository (GEO) at https://www.ncbi.nlm.nih.gov/geo/, reference number GSE206244.

## References

[ppl13771-bib-0001] Ahmed, M.F.A. (2018) Evaluation of some biocontrol agents to control Thompson seedless grapevine powdery mildew disease. Egyptian Journal of Biological Pest Control, 28, 2–7.

[ppl13771-bib-0002] Ali, M. , Javaid, A. , Naqvi, S.H. , Batcho, A. , Kayani, W.K. , Lal, A. et al. (2020) Biotic stress triggered small RNA and RNAi defense response in plants. Molecular Biology Reports, 47, 5511–5522. 32562176 10.1007/s11033-020-05583-4

[ppl13771-bib-0003] Alonso, C. , Ramos‐Cruz, D. & Becker, C. (2019) The role of plant epigenetics in biotic interactions. New Phytologist, 221, 731–737. 30156271 10.1111/nph.15408 PMC6726468

[ppl13771-bib-0004] Anderson MJ (2017) Permutational multivariate analysis of variance (PERMANOVA). Wiley StatsRef: Statistics Reference Online 1–15

[ppl13771-bib-0005] Armijo, G. , Schlechter, R. , Agurto, M. , Muñoz, D. , Nuñez, C. & Arce‐Johnson, P. (2016) Grapevine pathogenic microorganisms: understanding infection strategies and host response scenarios. Frontiers in Plant Science, 7, 1–18. 27066032 10.3389/fpls.2016.00382 PMC4811896

[ppl13771-bib-0006] Ashapkin, V.V. , Kutueva, L.I. , Aleksandrushkina, N.I. & Vanyushin, B.F. (2020) Epigenetic mechanisms of plant adaptation to biotic and abiotic stresses. International Journal of Molecular Sciences, 21, 1–32. 10.3390/ijms21207457 PMC7589735 33050358

[ppl13771-bib-0007] Atighi, M.R. , Verstraeten, B. , Meyer, T.D. & Kyndt, T. (2020) Genome‐wide DNA hypomethylation shapes nematode pattern‐triggered immunity in plants. New Phytologist, 227, 545–558. 32162327 10.1111/nph.16532 PMC7317725

[ppl13771-bib-0008] Barozai, M.Y.K. & Aziz, A.N. (2018) Recent plant growth and stress management related significant advancements in epigenetics. Annals of Agrarian Science, 16, 416–421.

[ppl13771-bib-0009] Berr, A. , Ménard, R. , Heitz, T. & Shen, W.H. (2012) Chromatin modification and remodelling: a regulatory landscape for the control of Arabidopsis defence responses upon pathogen attack. Cellular Microbiology, 14, 829–839. 22405188 10.1111/j.1462-5822.2012.01785.x

[ppl13771-bib-0010] Berriri, S. , Gangappa, S.N. & Kumar, S.V. (2016) SWR1 chromatin‐remodeling complex subunits and H2A.Z have non‐overlapping functions in immunity and gene regulation in Arabidopsis. Molecular Plant, 9, 1051–1065. 27131447 10.1016/j.molp.2016.04.003 PMC4938710

[ppl13771-bib-0011] Bindea, G. , Mlecnik, B. , Hackl, H. , Charoentong, P. , Tosolini, M. , Kirilovsky, A. et al. (2009) ClueGO: a cytoscape plug‐in to decipher functionally grouped gene ontology and pathway annotation networks. Bioinformatics, 25, 1091–1093. 19237447 10.1093/bioinformatics/btp101 PMC2666812

[ppl13771-bib-0012] Boso, S. , Alonso‐Villaverde, V. , Gago, P. , Santiago, J.L. & Martínez, M.C. (2014) Susceptibility to downy mildew (*Plasmopara viticola*) of different Vitis varieties. Crop Protection, 63, 26–35.

[ppl13771-bib-0013] Boso, S. & Kassemeyer, H.H. (2008) Different susceptibility of European grapevine cultivars for downy mildew. Vitis: Journal of Grapevine Research, 47, 39–49.

[ppl13771-bib-0014] Bouyer, D. , Heese, M. , Chen, P. , Harashima, H. , Roudier, F. , Grüttner, C. et al. (2018) Genome‐wide identification of RETINOBLASTOMA RELATED 1 binding sites in Arabidopsis reveals novel DNA damage regulators. PLoS Genetics, 14, 1–35. 10.1371/journal.pgen.1007797 PMC6268010 30500810

[ppl13771-bib-0015] Boyko, A. & Kovalchuk, I. (2008) Epigenetic control of plant stress response. Environmental and Molecular Mutagenesis, 49, 61–72. 17948278 10.1002/em.20347

[ppl13771-bib-0016] Breitling, R. , Armengaud, P. , Amtmann, A. & Herzyk, P. (2004) Rank products: a simple, yet powerful, new method to detect differentially regulated genes in replicated microarray experiments. FEBS Letters, 573, 83–92. 15327980 10.1016/j.febslet.2004.07.055

[ppl13771-bib-0017] Brilli, M. , Asquini, E. , Moser, M. , Bianchedi, P.L. , Perazzolli, M. & Si‐Ammour, A. (2018) A multi‐omics study of the grapevine‐downy mildew (*Plasmopara viticola*) pathosystem unveils a complex protein coding‐ and noncoding‐based arms race during infection. Scientific Reports, 8, 1–12. 29335535 10.1038/s41598-018-19158-8 PMC5768699

[ppl13771-bib-0018] Brocklehurst, S. , Watson, M. , Carr, I.M. , Out, S. , Heidmann, I. & Meyer, P. (2018) Induction of epigenetic variation in Arabidopsis by over‐expression of DNA METHYLTRANSFERASE1 (MET1). PLoS One, 13, 1–22. 10.1371/journal.pone.0192170 PMC5821449 29466369

[ppl13771-bib-0019] Buonassisi, D. , Colombo, M. , Migliaro, D. , Dolzani, C. , Peressotti, E. , Mizzotti, C. et al. (2017) Breeding for grapevine downy mildew resistance: a review of “omics” approaches. Euphytica, 213, 1–21.

[ppl13771-bib-0020] Camborde, L. , Raynaud, C. , Dumas, B. & Gaulin, E. (2019) DNA‐damaging effectors: new players in the effector arena. Trends in Plant Science, 24, 1094–1101. 31699522 10.1016/j.tplants.2019.09.012

[ppl13771-bib-0021] Catacchio, C.R. , Alagna, F. , Perniola, R. , Bergamini, C. , Rotunno, S. , Calabrese, F.M. et al. (2019) Transcriptomic and genomic structural variation analyses on grape cultivars reveal new insights into the genotype‐dependent responses to water stress. Scientific Reports, 9, 1–15. 30809001 10.1038/s41598-019-39010-x PMC6391451

[ppl13771-bib-0022] Chan, C. & Zimmerli, L. (2019) The histone demethylase IBM1 positively regulates Arabidopsis immunity by control of defense gene expression. Frontiers in Plant Science, 10, 1–10. 31956325 10.3389/fpls.2019.01587 PMC6951416

[ppl13771-bib-0023] Chen, H. , Shu, H. , Wang, L. , Zhang, F. , Li, X. , Ochola, S.O. et al. (2018) Phytophthora methylomes are modulated by 6mA methyltransferases and associated with adaptive genome regions. Genome Biology, 19, 1–16. 30382931 10.1186/s13059-018-1564-4 PMC6211444

[ppl13771-bib-0024] Chen, J. , Clinton, M. , Qi, G. , Wang, D. , Liu, F. & Qing, F.Z. (2020) Reprogramming and remodeling: transcriptional and epigenetic regulation of salicylic acid‐mediated plant defense. Journal of Experimental Botany, 71, 5256–5268. 32060527 10.1093/jxb/eraa072

[ppl13771-bib-0025] Chong, J. , Le Henanff, G. , Bertsch, C. & Walter, B. (2008) Identification, expression analysis and characterization of defense and signaling genes in *Vitis vinifera* . Plant Physiology and Biochemistry, 46, 469–481. 17988883 10.1016/j.plaphy.2007.09.010

[ppl13771-bib-0026] Coolen, S. , Van Pelt, J.A. , Van Wees, S.C.M. & Pieterse, C.M.J. (2019) Mining the natural genetic variation in Arabidopsis thaliana for adaptation to sequential abiotic and biotic stresses. Planta, 249, 1087–1105. 30547240 10.1007/s00425-018-3065-9

[ppl13771-bib-0027] Cui, N. , Chen, X. , Shi, Y. , Chi, M. , Hu, J. , Lai, K. et al. (2021) Changes in the epigenome and transcriptome of rice in response to Magnaporthe oryzae infection. Crop Journal, 9, 843–853.

[ppl13771-bib-0028] De‐La‐Peña, C. , Rangel‐Cano, A. & Alvarez‐Venegas, R. (2012) Regulation of disease‐responsive genes mediated by epigenetic factors: interaction of Arabidopsis‐pseudomonas. Molecular Plant Pathology, 13, 388–398. 22023111 10.1111/j.1364-3703.2011.00757.x PMC6638851

[ppl13771-bib-0029] Deleris, A. , Halter, T. & Navarro, L. (2016) DNA methylation and demethylation in plant immunity. Annual Review of Phytopathology, 54, 579–603. 10.1146/annurev-phyto-080615-100308 27491436

[ppl13771-bib-0030] Desvoyes, B. & Gutierrez, C. (2020) Roles of plant retinoblastoma protein: cell cycle and beyond. The EMBO Journal, 39, 1–18. 10.15252/embj.2020105802 PMC7527812 32865261

[ppl13771-bib-0031] Ding, B. & Wang, G.L. (2015) Chromatin versus pathogens: the function of epigenetics in plant immunity. Frontiers in Plant Science, 6, 1–8. 26388882 10.3389/fpls.2015.00675 PMC4557108

[ppl13771-bib-0032] Dowen, R.H. , Pelizzola, M. , Schmitz, R.J. , Lister, R. , Dowen, J.M. , Nery, J.R. et al. (2012) Widespread dynamic DNA methylation in response to biotic stress. Proceedings of the National Academy of Sciences of the United States of America, 109, E2183–E2191. 22733782 10.1073/pnas.1209329109 PMC3420206

[ppl13771-bib-0033] Eisenmann, B. , Czemmel, S. , Ziegler, T. , Buchholz, G. , Kortekamp, A. , Trapp, O. et al. (2019) Rpv3‐1 mediated resistance to grapevine downy mildew is associated with specific host transcriptional responses and the accumulation of stilbenes. BMC Plant Biology, 19, 1–17. 31387524 10.1186/s12870-019-1935-3 PMC6685164

[ppl13771-bib-0034] Elhamamsy, A.R. (2016) DNA methylation dynamics in plants and mammals: overview of regulation and dysregulation. Cell Biochemistry and Function, 34, 289–298. 27003927 10.1002/cbf.3183

[ppl13771-bib-0035] Erdmann, R.M. & Picard, C.L. (2020) RNA‐directed DNA methylation. PLoS Genetics, 16, 1–31. 10.1371/journal.pgen.1009034 PMC7544125 33031395

[ppl13771-bib-0036] Espinas, N.A. , Saze, H. & Saijo, Y. (2016) Epigenetic control of defense signaling and priming in plants. Frontiers in Plant Science, 7, 1–7. 27563304 10.3389/fpls.2016.01201 PMC4980392

[ppl13771-bib-0037] Figueiredo, A. , Figueiredo, J. , Maia, M. , Cavaco, A.R. , Laureano, G. , Nascimento, R. et al. (2018) Subtilisin‐like proteases and lipid signalling events play an important role in grapevine resistance to downy mildew: a systems biology approach. Revista de Ciências Agrárias, 41, 61–66.

[ppl13771-bib-0038] Figueiredo, A. , Fortes, A.M. , Ferreira, S. , Sebastiana, M. , Choi, Y.H. , Sousa, L. et al. (2008) Transcriptional and metabolic profiling of grape (*Vitis vinifera* L.) leaves unravel possible innate resistance against pathogenic fungi. Journal of Experimental Botany, 59, 3371–3381. 18648103 10.1093/jxb/ern187

[ppl13771-bib-0039] Figueiredo, A. , Martins, J. , Sebastiana, M. , Guerreiro, A. , Silva, A. , Matos, A.R. et al. (2017) Specific adjustments in grapevine leaf proteome discriminating resistant and susceptible grapevine genotypes to *Plasmopara viticola* . Journal of Proteomics, 152, 48–57. 27989945 10.1016/j.jprot.2016.10.012

[ppl13771-bib-0040] Figueiredo, A. , Monteiro, F. , Fortes, A.M. , Bonow‐Rex, M. , Zyprian, E. , Sousa, L. et al. (2012) Cultivar‐specific kinetics of gene induction during downy mildew early infection in grapevine. Functional and Integrative Genomics, 12, 379–386. 22246600 10.1007/s10142-012-0261-8

[ppl13771-bib-0041] Fröbel, S. & Zyprian, E. (2019) Colonization of different grapevine tissues by *Plasmopara viticola*: a histological study. Frontiers in Plant Science, 10, 1–13. 31396252 10.3389/fpls.2019.00951 PMC6667660

[ppl13771-bib-0042] Gallusci, P. , Dai, Z. , Génard, M. , Gauffretau, A. , Leblanc‐Fournier, N. , Richard‐Molard, C. et al. (2017) Epigenetics for plant improvement: current knowledge and modeling avenues. Trends in Plant Science, 22, 610–623. 28587758 10.1016/j.tplants.2017.04.009

[ppl13771-bib-0043] Geng, S. , Kong, X. , Song, G. , Jia, M. , Guan, J. , Wang, F. et al. (2019) DNA methylation dynamics during the interaction of wheat progenitor *Aegilops tauschii* with the obligate biotrophic fungus *Blumeria graminis* f. sp. tritici. New Phytologist, 221, 1023–1035. 30256420 10.1111/nph.15432 PMC6586159

[ppl13771-bib-0044] He, C. , Zhang, Z. , Li, B. & Tian, S. (2020) The pattern and function of DNA methylation in fungal plant pathogens. Microorganisms, 8, 1–14. 10.3390/microorganisms8020227 PMC7074731 32046339

[ppl13771-bib-0045] He, S. , Yuan, G. , Bian, S. , Han, X. , Liu, K. , Cong, P. et al. (2020) Major latex protein MdMLP423 negatively regulates defense against fungal infections in apple. International Journal of Molecular Sciences, 21, 1–20. 10.3390/ijms21051879 PMC7084931 32164313

[ppl13771-bib-0046] Hewezi, T. , Pantalone, V. , Bennett, M. , Neal Stewart, C. & Burch‐Smith, T.M. (2018) Phytopathogen‐induced changes to plant methylomes. Plant Cell Reports, 37, 17–23. 28756583 10.1007/s00299-017-2188-y

[ppl13771-bib-0047] Hoang, T.V. , Vo, K.T.X. , Hong, W.J. , Jung, K.H. & Jeon, J.S. (2018) Defense response to pathogens through epigenetic regulation in Rice. Journal of Plant Biology, 61, 1–10.

[ppl13771-bib-0048] Howe, E. , Holton, K. , Nair, S. , Schlauch, D. , Sinha, R. & Quackenbush, J. (2010) MeV: MultiExperiment Viewer. In: Ochs M , Casagrande J , Davuluri R , (eds), Biomedical Informatics for Cancer Research, Boston, MA: Springer, pp. 267–277.

[ppl13771-bib-0049] Huang, C.Y. & Jin, H. (2022) Coordinated epigenetic regulation in plants: a potent managerial tool to conquer biotic stress. Frontiers in Plant Science, 12, 1–11. 10.3389/fpls.2021.795274 PMC8762163 35046981

[ppl13771-bib-0050] Iqbal, Z. , Iqbal, M.S. , Hashem, A. , Abd_Allah, E.F. & Ansari, M.I. (2021) Plant defense responses to biotic stress and its interplay with fluctuating dark/light conditions. Frontiers in Plant Science, 12, 1–22. 10.3389/fpls.2021.631810 PMC7982811 33763093

[ppl13771-bib-0051] Juliá, M. , Telenti, A. & Rausell, A. (2015) Sincell: an R/Bioconductor package for statistical assessment of cell‐state hierarchies from single‐cell RNA‐seq. Bioinformatics, 31, 3380–3382. 26099264 10.1093/bioinformatics/btv368 PMC4595899

[ppl13771-bib-0052] Kim, J.H. (2021) Multifaceted chromatin structure and transcription changes in plant stress response. International Journal of Molecular Sciences, 22, 1–25. 10.3390/ijms22042013 PMC7922328 33670556

[ppl13771-bib-0053] Köhler, C. & Springer, N. (2017) Plant epigenomics: deciphering the mechanisms of epigenetic inheritance and plasticity in plants. Genome Biology, 18, 1–3. 28683755 10.1186/s13059-017-1260-9 PMC5501107

[ppl13771-bib-0054] Kong, L. , Liu, Y. , Wang, X. & Chang, C. (2020) Insight into the role of epigenetic processes in abiotic and biotic stress response in wheat and barley. International Journal of Molecular Sciences, 21, 1–15. 10.3390/ijms21041480 PMC7073019 32098241

[ppl13771-bib-0055] Kruskal, W.H. & Wallis, W.A. (1952) Use of ranks in one‐criterion variance analysis Author (Kruskal–Wallis test). Journal of the American Statistical Association, 47, 583–621.

[ppl13771-bib-0056] Kumar, V. , Thakur, J.K. & Prasad, M. (2021) Histone acetylation dynamics regulating plant development and stress responses. Cellular and Molecular Life Sciences, 78, 4467–4486. 33638653 10.1007/s00018-021-03794-x PMC11072255

[ppl13771-bib-0057] Kuźnicki, D. , Meller, B. , Arasimowicz‐Jelonek, M. , Braszewska‐Zalewska, A. , Drozda, A. & Floryszak‐Wieczorek, J. (2019) BABA‐induced DNA methylome adjustment to intergenerational defense priming in potato to Phytophthora infestans. Frontiers in Plant Science, 10, 1–16. 31214209 10.3389/fpls.2019.00650 PMC6554679

[ppl13771-bib-0058] Lämke, J. & Bäurle, I. (2017) Epigenetic and chromatin‐based mechanisms in environmental stress adaptation and stress memory in plants. Genome Biology, 18, 1–11. 28655328 10.1186/s13059-017-1263-6 PMC5488299

[ppl13771-bib-0059] Laureano, G. , Figueiredo, J. , Cavaco, A.R. , Duarte, B. , Caçador, I. , Malhó, R. et al. (2018) The interplay between membrane lipids and phospholipase A family members in grapevine resistance against *Plasmopara viticola* . Scientific Reports, 8, 1–15. 30266912 10.1038/s41598-018-32559-z PMC6162203

[ppl13771-bib-0060] Laurell, C. , Berglund, T. & Ohlsson, A.B. (2022) Transcriptome analysis shows nicotinamide seed treatment alters expression of genes involved in defense and epigenetic processes in roots of seedlings of *Picea abies* . Journal of Forestry Research, 33, 1365–1375.

[ppl13771-bib-0061] Lee, C.H. & Carroll, B.J. (2018) Evolution and diversification of small RNA pathways in flowering plants. Plant & Cell Physiology, 59, 2169–2187. 30169685 10.1093/pcp/pcy167

[ppl13771-bib-0062] Leonetti, P. & Molinari, S. (2020) Epigenetic and metabolic changes in root‐knot nematode‐plant interactions. International Journal of Molecular Sciences, 21, 1–17. 10.3390/ijms21207759 PMC7589425 33092207

[ppl13771-bib-0063] Lievens, L. , Pollier, J. , Goossens, A. , Beyaert, R. & Staal, J. (2017) Abscisic acid as pathogen effector and immune regulator. Frontiers in Plant Science, 8, 1–15. 28469630 10.3389/fpls.2017.00587 PMC5395610

[ppl13771-bib-0064] Liu, J. , Zhi, P. , Wang, X. , Fan, Q. & Chang, C. (2019) Wheat WD40‐repeat protein TaHOS15 functions in a histone deacetylase complex to fine‐tune defense responses to Blumeria graminis f.sp. tritici. Journal of Experimental Botany, 70, 255–268. 30204899 10.1093/jxb/ery330

[ppl13771-bib-0065] Liu, L. , Zhang, B. , Wang, H. , Yu, S.Y. , Guan, T.S. , Huang, Y.F. et al. (2020) Candidate resistance genes selection and transcriptome analysis for the early responses to *Plasmopara viticola* infection in grape cultivars. Journal of Plant Pathology, 102, 857–869.

[ppl13771-bib-0066] Livak, K.J. & Schmittgen, T.D. (2001) Analysis of relative gene expression data using real‐time quantitative PCR and the 2(‐Delta Delta C(T)) method. Methods (San Diego, Calif), 25, 402–408. 11846609 10.1006/meth.2001.1262

[ppl13771-bib-0067] Lo Scalzo, R. , Fibiani, M. , Pietromarchi, P. , Mandalà, C. & La Torre, A. (2012) Effects of different fungicide treatments on grape, must and wine quality. Communications in Agricultural and Applied Biological Sciences, 77, 151–161. 23878969

[ppl13771-bib-0068] López, A. , Ramírez, V. , García‐Andrade, J. , Flors, V. & Vera, P. (2011) The RNA silencing enzyme RNA polymerase V is required for plant immunity. PLoS Genetics, 7, 1–10. 10.1371/journal.pgen.1002434 PMC3248562 22242006

[ppl13771-bib-0069] López Sánchez, A. , Stassen, J.H.M. , Furci, L. , Smith, L.M. & Ton, J. (2016) The role of DNA (de)methylation in immune responsiveness of Arabidopsis. Plant Journal, 88, 361–374. 10.1111/tpj.13252 PMC5132069 27341062

[ppl13771-bib-0070] Maia, M. , Maccelli, A. , Nascimento, R. , Ferreira, A.E.N. , Crestoni, M.E. , Cordeiro, C. et al. (2019) Early detection of *Plasmopara viticola*‐infected leaves through FT‐ICR‐MS metabolic profiling. Acta Horticulturae, 1248, 575–580.

[ppl13771-bib-0071] March‐Díaz, R. , García‐Domínguez, M. , Lozano‐Juste, J. , León, J. , Florencio, F.J. & Reyes, J.C. (2008) Histone H2A.Z and homologues of components of the SWR1 complex are required to control immunity in Arabidopsis. Plant Journal, 53, 475–487. 10.1111/j.1365-313X.2007.03361.x 17988222

[ppl13771-bib-0072] Marsico, A.D. , Perniola, R. & Bergamini, C. (2018) Nuovi genotipi di V. vinifera poco sensibili a Peronospora ottenuti dal Crea. Rivista di frutticoltura e di ortofloricoltura, 82, 26–31.

[ppl13771-bib-0073] Nascimento, R. , Maia, M. , Ferreira, A.E.N. , Silva, A.B. , Freire, A.P. , Cordeiro, C. et al. (2019) Early stage metabolic events associated with the establishment of *Vitis vinifera*: *Plasmopara viticola* compatible interaction. Plant Physiology and Biochemistry, 137, 1–13. 30710794 10.1016/j.plaphy.2019.01.026

[ppl13771-bib-0074] OIV (2009) 2nd Edition of the OIV Descriptor List for Grape Varieties and Vitis Species. Available from: https://www.oiv.int/en/technical-standards-and-documents/ (accessed July 2019)

[ppl13771-bib-0075] OIV (2021) State of the world vitivinicultural sector in 2020. *International Organisation of Vine and Wine* 1–19.

[ppl13771-bib-0076] Panigrahi, G.K. , Sahoo, A. & Satapathy, K.B. (2021) Insights to plant immunity: defense signaling to epigenetics. Physiological and Molecular Plant Pathology, 113, 1–7.

[ppl13771-bib-0077] Parker, A.H. , Wilkinson, S.W. & Ton, J. (2021) Epigenetics: a catalyst of plant immunity against pathogens. New Phytologist, 233, 66–83. 34455592 10.1111/nph.17699

[ppl13771-bib-0078] Pereira, G. , Pereira, J. , Santos, R.B. & Figueiredo, A. (2022) Uncovering the role of DNA methyltransferases in grapevine: *Plasmopara viticola* interaction—from genome‐wide characterization to global methylation patterns. Gene, 837, 1–9. 10.1016/j.gene.2022.146693 35738444

[ppl13771-bib-0079] Polesani, M. , Bortesi, L. , Ferrarini, A. et al. (2010) General and species‐specific transcriptional responses to downy mildew infection in a susceptible (*Vitis vinifera*) and a resistant (*V. riparia*) grapevine species. BMC Genomics, 11, 117. 20167053 10.1186/1471-2164-11-117 PMC2831845

[ppl13771-bib-0080] Prakash, V. , Devendran, R. & Chakraborty, S. (2017) Overview of plant RNA dependent RNA polymerases in antiviral defense and gene silencing. Indian Journal of Plant Physiology, 22, 493–505.

[ppl13771-bib-0081] Pujara, D.S. , Kim, S.‐I. , Nam, J.C. , Mayorga, J. , Elmore, I. , Kumar, M. et al. (2021) Imaging‐based resistance assay using enhanced luminescence‐tagged pseudomonas syringae reveals a complex epigenetic network in plant defense signaling pathways. Molecular Plant‐Microbe Interactions, 34, 990–1000. 34010013 10.1094/MPMI-12-20-0351-TA

[ppl13771-bib-0082] Qin, C. , Li, B. , Fan, Y. , Zhang, X. , Yu, Z. , Ryabov, E. et al. (2017) Roles of dicer‐like proteins 2 and 4 in intra‐ and intercellular antiviral silencing. Plant Physiology, 174, 1067–1081. 28455401 10.1104/pp.17.00475 PMC5462052

[ppl13771-bib-0083] Ramirez‐Prado, J.S. , Abulfaraj, A.A. , Rayapuram, N. , Benhamed, M. & Hirt, H. (2018) Plant immunity: from signaling to epigenetic control of defense. Trends in Plant Science, 23, 833–844. 29970339 10.1016/j.tplants.2018.06.004

[ppl13771-bib-0084] Ramirez‐Prado, J.S. , Piquerez, S.J.M. , Bendahmane, A. , Hirt, H. , Raynaud, C. & Benhamed, M. (2018) Modify the histone to win the battle: chromatin dynamics in plant–pathogen interactions. Frontiers in Plant Science, 9, 1–18. 29616066 10.3389/fpls.2018.00355 PMC5868138

[ppl13771-bib-0085] Ramos‐Cruz, D. , Troyee, A.N. & Becker, C. (2021) Epigenetics in plant organismic interactions. Current Opinion in Plant Biology, 61, 1–10. 10.1016/j.pbi.2021.102060 34087759

[ppl13771-bib-0086] Rojas‐Rojas, F.U. & Vega‐Arreguín, J.C. (2021) Epigenetic insight into regulatory role of chromatin covalent modifications in lifecycle and virulence of Phytophthora. Environmental Microbiology Reports, 13, 445–457. 33876568 10.1111/1758-2229.12954

[ppl13771-bib-0087] Santos, R.B. , Nascimento, R. , Coelho, A.V. & Figueiredo, A. (2020) Grapevine: downy mildew rendezvous—proteome analysis of the first hours of an incompatible interaction. Plants, 9, 1–17. 10.3390/plants9111498 PMC7694532 33167573

[ppl13771-bib-0088] Savary, S. , Delbac, L. , Rochas, A. , Taisant, G. & Willocquet, L. (2009) Analysis of nonlinear relationships in dual epidemics, and its application to the management of grapevine downy and powdery mildews. Phytopathology, 99, 930–942. 19594312 10.1094/PHYTO-99-8-0930

[ppl13771-bib-0089] Shannon, P. , Markiel, A. , Ozier, O. , Baliga, N.S. , Wang, J.T. , Ramage, D. et al. (2013) Cytoscape: a software environment for integrated models. Genome Research, 13, 2498–2504. 10.1101/gr.1239303 PMC403769 14597658

[ppl13771-bib-0090] Sheikh, A.H. , Nawaz, K. , Tabassum, N. , Trapp, M. , Alhoraibi, H. , Rayapuram, N. et al. (2022) Linker histone H1 regulates defense priming and immunity in plants. bioRxiv. Available from: 10.1101/2022.04.11.487821 PMC10201415 36840717

[ppl13771-bib-0091] Song, J. & Bent, A.F. (2014) Microbial pathogens trigger host DNA double‐strand breaks whose abundance is reduced by plant defense responses. PLoS Pathogens, 10, 1–11. 10.1371/journal.ppat.1004030 PMC3974866 24699527

[ppl13771-bib-0092] Song, Z.T. , Liu, J.X. & Han, J.J. (2021) Chromatin remodeling factors regulate environmental stress responses in plants. Journal of Integrative Plant Biology, 63, 438–450. 33421288 10.1111/jipb.13064

[ppl13771-bib-0093] Unger, S. , Büche, C. , Boso, S. & Kassemeyer, H.‐H. (2007) The course of colonization of two different Vitis genotypes by *Plasmopara viticola* indicates compatible and incompatible host‐pathogen interactions. Phytopathology, 97, 780–786. 18943926 10.1094/PHYTO-97-7-0780

[ppl13771-bib-0094] Vezzulli, S. , Vecchione, A. , Stefanini, M. & Zulini, L. (2018) Downy mildew resistance evaluation in 28 grapevine hybrids promising for breeding programs in Trentino region (Italy). European Journal of Plant Pathology, 150, 485–495.

[ppl13771-bib-0095] Wang, L. , Chen, H. , Li, J. , Shu, H. , Zhang, X. , Wang, Y. et al. (2020) Effector gene silencing mediated by histone methylation underpins host adaptation in an oomycete plant pathogen. Nucleic Acids Research, 48, 1790–1799. 31819959 10.1093/nar/gkz1160 PMC7039004

[ppl13771-bib-0096] Wang, M. , Weiberg, A. , Lin, F.M. , Thomma, B.P.H.J. , Da, H.H. & Jin, H. (2016) Bidirectional cross‐kingdom RNAi and fungal uptake of external RNAs confer plant protection. Nature Plants, 2, 1–10. 10.1038/nplants.2016.151 PMC5040644 27643635

[ppl13771-bib-0097] Wang, S. , Durrant, W.E. , Song, J. , Spivey, N.W. & Dong, X. (2010) Arabidopsis BRCA2 and RAD51 proteins are specifically involved in defense gene transcription during plant immune responses. Proceedings of the National Academy of Sciences of the United States of America, 107, 22716–22721. 21149701 10.1073/pnas.1005978107 PMC3012525

[ppl13771-bib-0098] Wang, S. , Gu, Y. , Zebell, S.G. , Anderson, L.K. , Wang, W. , Mohan, R. et al. (2014) A noncanonical role for the CKI‐RB‐E2F cell‐cycle signaling pathway in plant effector‐triggered immunity. Cell Host and Microbe, 16, 787–794. 25455564 10.1016/j.chom.2014.10.005 PMC4282163

[ppl13771-bib-0099] Weiberg, A. , Wang, M. , Lin, F.M. , Zhao, H. , Zhang, Z. , Kaloshian, I. et al. (2013) Fungal small RNAs suppress plant immunity by hijacking host RNA interference pathways. Science, 342, 118–123. 24092744 10.1126/science.1239705 PMC4096153

[ppl13771-bib-0100] Xiao, D. , Zhou, K. , Yang, X. , Yang, Y. , Ma, Y. & Wang, Y. (2021) Crosstalk of DNA methylation triggered by pathogen in poplars with different resistances. Frontiers in Microbiology, 12, 1–15. 10.3389/fmicb.2021.750089 PMC8748266 35027912

[ppl13771-bib-0102] Xie, H. , Konate, M. , Sai, N. , Tesfamicael, K.G. , Cavagnaro, T. , Gilliham, M. et al. (2017) Global DNA methylation patterns can play a role in defining terroir in grapevine (*Vitis vinifera* cv. Shiraz). Frontiers in Plant Science, 8, 1–16. 29163587 10.3389/fpls.2017.01860 PMC5670326

[ppl13771-bib-0103] Yan, L. , Fan, G. & Li, X. (2019) Genome‐wide analysis of three histone marks and gene expression in Paulownia fortunei with phytoplasma infection. BMC Genomics, 20, 1–14. 30898112 10.1186/s12864-019-5609-1 PMC6429711

[ppl13771-bib-0104] Zhi, P. & Chang, C. (2021) Exploiting epigenetic variations for crop disease resistance improvement. Frontiers in Plant Science, 12, 1–13. 10.3389/fpls.2021.692328 PMC8212930 34149790

[ppl13771-bib-0105] Zhu, Q.‐H. , Shan, W.‐X. , Ayliffe, M.A. & Wang, M.‐B. (2016) Epigenetic mechanisms: an emerging player in plant‐microbe interactions. Molecular Plant‐Microbe Interactions, 29, 187–196. 26524162 10.1094/MPMI-08-15-0194-FI

