# Supplementary material for: Transcriptomic and methylation analysis of susceptible and tolerant grapevine genotypes following Plasmopara viticola infection
Source: Physiol Plant. 2022 Sep 21;174(5):e13771. doi: 10.1111/ppl.13771 (PMC9826190; doi:10.1111/ppl.13771)
Supplement: Supplementary file 1 — Appendix S1. Supporting information. [file PPL-174-0-s001.pdf]

SUPPORTING INFORMATION

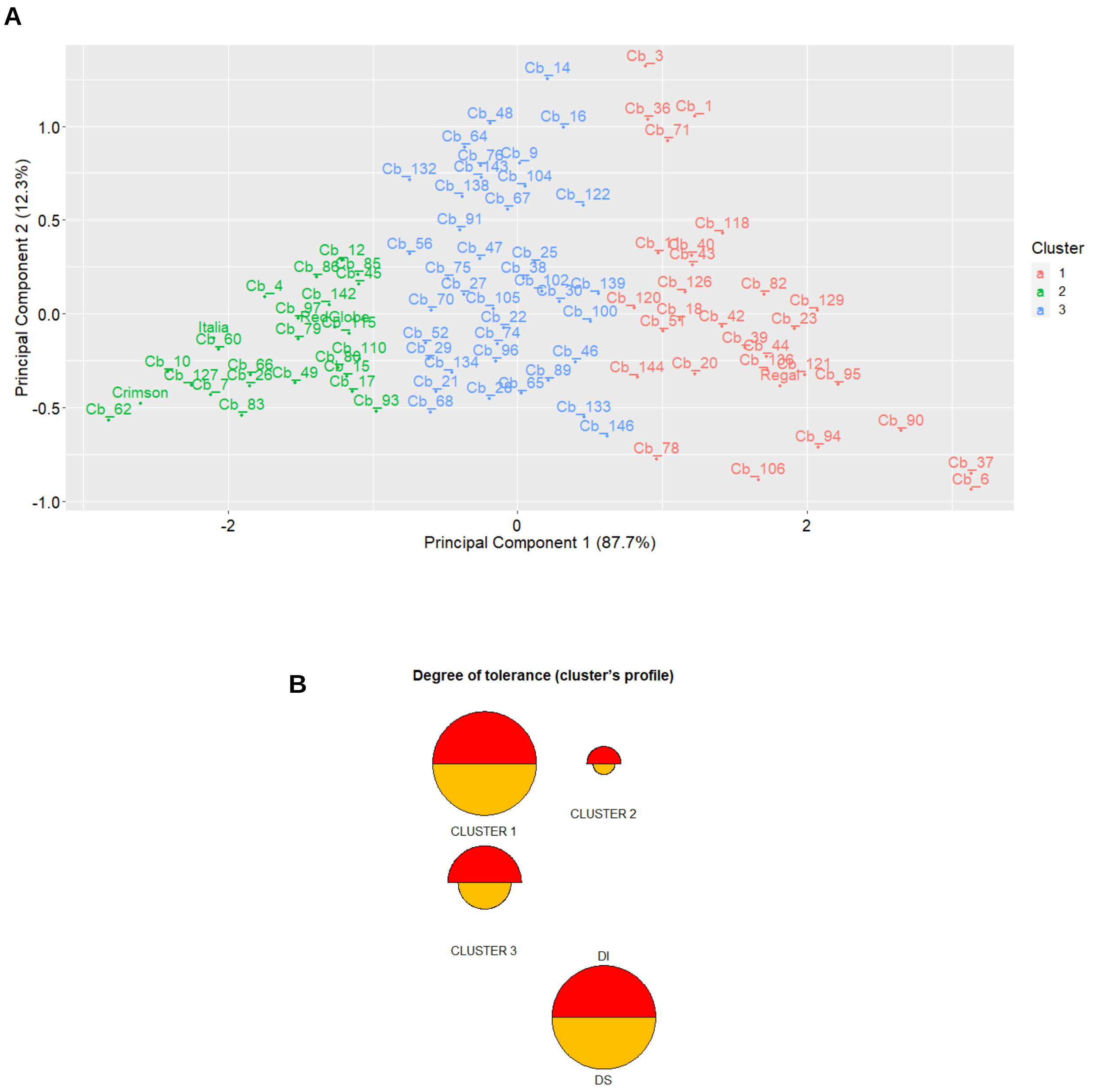

Figure S1. Degree of tolerance/susceptibility to *P. viticola* within the cross population Red Globe x Regal. Cluster analysis (k-means) (A) and cluster profile (B) perfomed on 115 new genotypes (Red Globe x Regal seedless) based on their tolerance/susceptibility to *P. viticola* infections on grape bunches. Genotypes belonging to C1 cluster showed higher susceptibility to *P. viticola*, compared to C3 (middle susceptibility) and C2 (lower susceptibility). Cb_12 = N20/012 (C2); Cb_18 = N20/020 (C1); Cb_20 = N20/029 (C1); Cb_115 = N23/018 (C2). DI = Disease index; DS = Disease severity. Circle dimension is proportional to indices values.

A

Gene	Gene ID	Fold change microarray	Fold change RT-qPCR
SYD (T24)	VIT_205s0020g02000	-4,5	-2,66
LYM (T6)	VIT_218s0001g03080	-2,5	-2,44
HOS15 (S6)	VIT_218s0001g09610	-2,8	-1,49
SPO11 (S24)	VIT_219s0015g00280	2,6	2,68
SPO11 (T6)	VIT_219s0015g00280	4,5	1,13
DCL2 (T24)	VIT_204s0023g00920	-4,1	-5,88
H2AX (T6)	VIT_207s0104g00960	-8,0	-5,88

Pearson correlation = 0,876749751

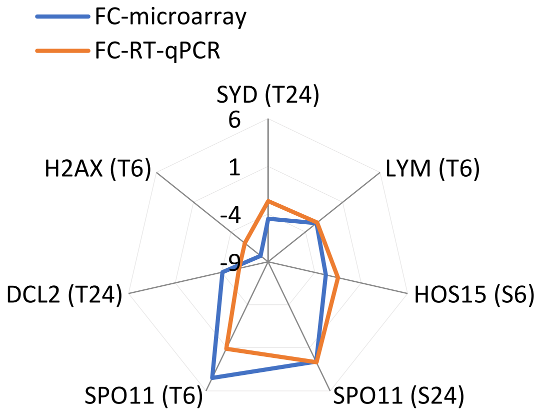

B

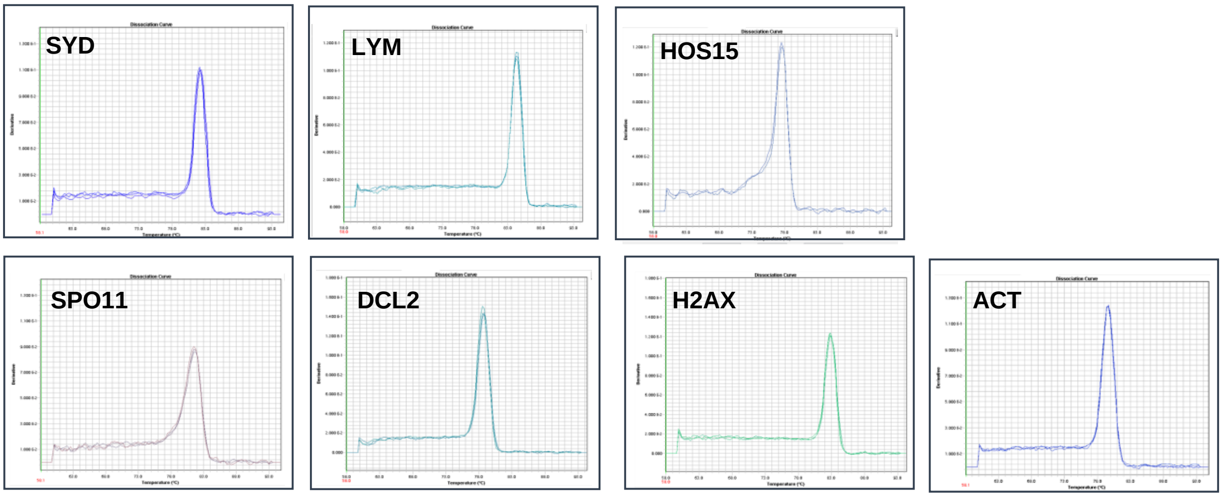

Figure S2. RT-qPCR analysis. A) Comparison of gene expression between microarray and RT-qPCR, calculated as fold change of *P. viticola* inoculated samples (leaves) vs control (mock). B) Dissociation curves of RT-qPCR analyses. SYD: Chromatin structure-remodeling complex protein SPLAYED; LYM: LysM domain-containing GPI-anchored protein 1; HOS15: F-box-like/WD repeat-containing protein TBL1X; SPO11: Meiotic recombination protein SPO11-1; DCL2: Dicer-like homolog 2; H2AX: Histone H2AX; ACT: Actin (housekeeping gene).

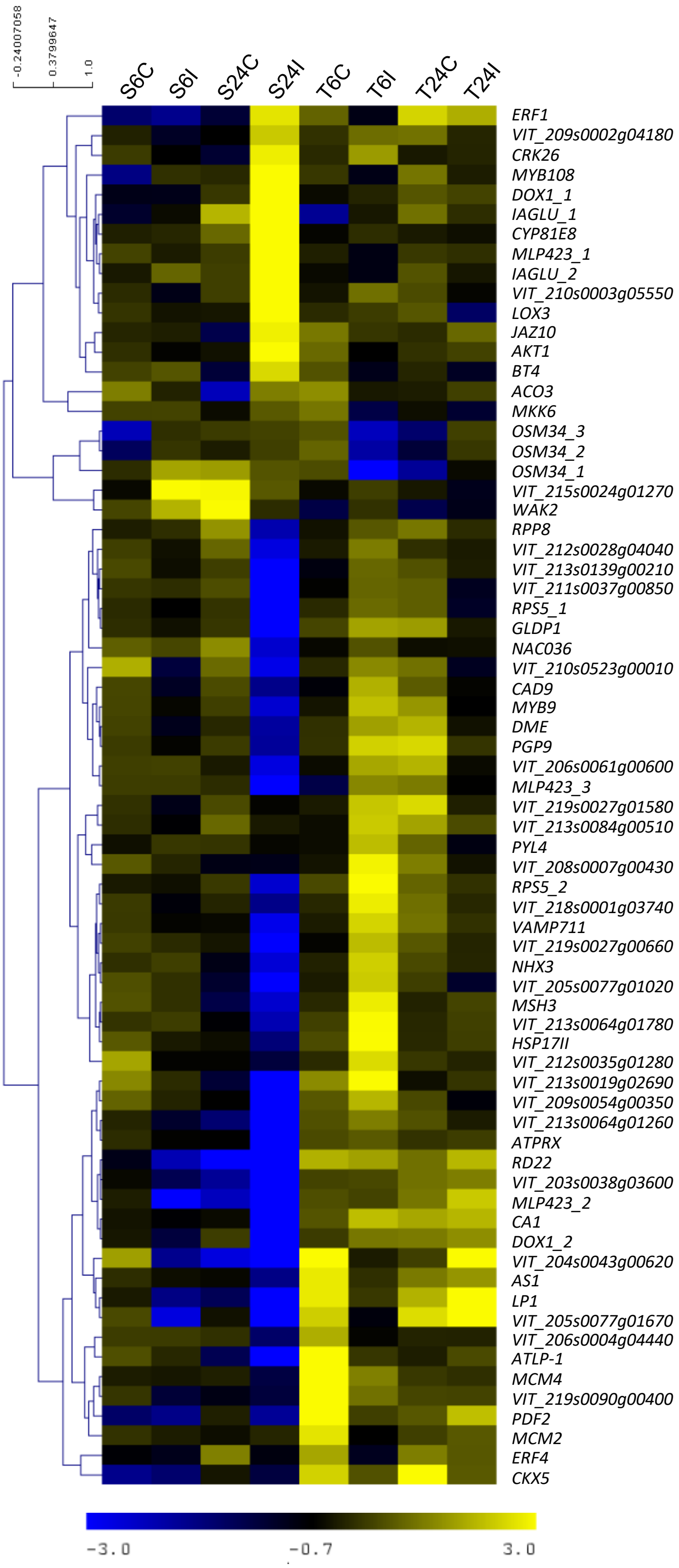

Figure S3. Hierarchical clustering of defense related genes (DR-DEGs) differentially expressed after *P. viticola* infection. Microarray expression data of the putatively susceptible (S) and tolerant (T) genotypes at 6 and 24 hours post inoculation (hpi) with different tratments, mock (C) and inoculated (I) were used for clustering analysis. A selection of genes significantly differentially expressed in the inoculated *versus* mock conditions is reported. For each gene, the Log_2 normalised expression value was used. The different colours indicate different expression values, as reported in the colour bar. Lowest and highest expression values are in blue and yellow, respectively. Gene IDs of the selected genes are reported in Table S4. A selection of defense gene is reported.

Table S1. Primers used for RT-qPCR analyses

Gene	Gene ID	Forward	Reverse
SYD	VIT_205s0020g02000	CCTCCTTCCCCTTCTGTTGC	TGAGCCTTCTGACTTTGCCC
LYM	VIT_218s0001g03080	TCCTAGTCCTTCGGTGGTGC	AGGGAAACTGGCTAAGGGGT
HOS15	VIT_218s0001g09610	ACCCTTGATTGGAATGGAGATGG	AAACAACATACCAACCCTGCTCC
SPO11	VIT_219s0015g00280	AGAGTTCCGGGTCATGGTCT	TCCTCACTTGGCATTTTCTCGT
DCL2	VIT_204s0023g00920	CGAGCATAAGAATAACACGCCCA	CCAAGCGAAGTGAAGCGAAC
H2AX	VIT_207s0104g00960	AAACTTCTGGGAGGCGTGAC	GATGCGGACCCAATCTCTCC

Table S4. Selection of defence related DEGs (DR-DEGs) with higher fold change regulation after *P. viticola* infection grouped through the pathway/gene they are related to.

GeneID V1	S6	S24	T6	T24	GeneName	Product	GenBank Acc.
**Pathogen-Associated Molecular Pattern (PAMP) triggered immunity**							
VIT_15s0024g01270	13,80				VIT_215s0024g01270	receptor-like protein kinase HSL1	XM_010662858.1
VIT_00s0429g00010		15,60			CRK26	PREDICTED: Vitis vinifera cysteine-rich receptor-like protein kinase 29	XM_010649338.2
VIT_03s0038g03600		-12,98			VIT_203s0038g03600	serine/threonine-protein kinase CDL1-like	XM_002279438.2
VIT_18s0041g00040	3,06	-12,69			WAK2	wall-associated receptor kinase 2-like	XM_010667126.1
VIT_18s0001g03740			7,59		VIT_218s0001g03740	PREDICTED: Vitis vinifera non-functional pseudokinase ZED1	XM_019217903.1
VIT_11s0016g02970			-5,21		MKK6	mitogen-activated protein kinase kinase 6	XM_002283455.3
**Jasmonic Acid pathway**							
VIT_01s0146g00480		18,76			JAZ10	protein tify 9	XM_002262714.2
VIT_00s0265g00170		25,45		-4,52	LOX3	lipoxygenase 2	
**Salicylic Acid pathway**							
VIT_07s0129g00210		13,22	-2,73		BT4	BTB/POZ and TAZ domain-containing protein 4	XM_003632444.2
VIT_12s0028g04040		-11,64			VIT_212s0028g04040	PREDICTED: Vitis vinifera protein ACCELERATED CELL DEATH 6-like	XM_019223359.1
VIT_14s0066g01670		10,09			DOX1_1	alpha-dioxygenase 1	XM_002279848.3
VIT_14s0066g01690		-12,93			DOX1_2	alpha-dioxygenase 1-like	XM_010662647.2
**Abscisic Acid pathway**							
VIT_04s0008g03950	-2,66	-18,41		2,07	RD22	dehydration-responsive protein rd22	
VIT_08s0058g00470			6,08		PYL4	abscisic acid receptor pyl4	XM_002264122.4
**Ethylene pathway**							
VIT_05s0049g00510		14,45	-3,16		ERF1	ethylene-responsive transcription factor 1B	XM_010651768.2
VIT_19s0014g02240			-6,67		ERF4	ethylene-responsive transcription factor 4	XP_002285146.1
VIT_05s0077g01020			6,10		VIT_205s0077g01020	probable N-acetyltransferase HLS1-like	XM_002272501.2
**Metabolism related Proteins**							
VIT_07s0129g00800		13,02			CYP81E8	isoflavone 2 -hydroxylase	XM_002283756.4
VIT_12s0059g02150	-2,57	11,76	-3,33		ACO3	aconitate hydratase cytoplasmic	XM_002279224.3
VIT_10s0003g05550		29,38			VIT_210s0003g05550	reticuline oxidase-like	XP_002269462.1
VIT_14s0066g01210		-30,10			CA1	PREDICTED: Vitis vinifera beta carbonic anhydrase 1, chloroplastic	XM_010662610.2
VIT_04s0008g01300		-19,02		-3,86	GLDP1	PREDICTED: Vitis vinifera glycine dehydrogenase (decarboxylating), mitochondrial	XM_010650056.1
VIT_04s0043g00620	-12,47	-10,43	-10,43	17,08	VIT_204s0043g00620	PREDICTED: Vitis vinifera GDSL esterase/lipase At5g33370-like	XM_010650646.2
**Pathogenesis-Related (PR) Proteins**							
VIT_05s0077g01690		17,35			MLP423_1	PREDICTED: Vitis vinifera pathogenesis-related protein 10.8 (PR10.8)	XM_002273779.4
VIT_05s0077g01670	-8,58	-10,23	-9,00		VIT_205s0077g01670	PREDICTED: Vitis vinifera pathogenesis-related protein 10.7 (PR10.7)	XM_002273754.4
VIT_01s0026g00570	-7,84	-54,44		2,34	MLP423_2	MLP-like protein 423	XM_002267183.3
VIT_13s0019g02690	-2,60	-11,73			VIT_213s0019g02690	putative lipid-transfer protein DIR1	XM_002281518.3
VIT_05s0077g01650		-8,90	6,31		MLP423_3	PREDICTED: Vitis vinifera pathogenesis-related protein 10.5	XM_002273946.4
VIT_17s0000g02470			-9,29		ATLP-1	thaumatin-like protein	XM_003634158.2
VIT_02s0025g04310	3,40		-12,99	2,87	OSM34_1	Vitis vinifera thaumatin-like protein (Tl3)	AF532965.1
VIT_02s0025g04330	3,11		-7,76	2,53	OSM34_2	Vitis vinifera VVTL1 (TL1), mRNA	NM_001281132.2
VIT_02s0025g04330	5,14		-7,66	3,80	OSM34_3	Vitis vinifera VVTL1 (TL1), mRNA	NM_001281132.2
VIT_06s0004g04440			-5,59		VIT_206s0004g04440	osmotin-like protein	XP_002281193.1
VIT_08s0058g01210	-2,96		-6,20	2,16	LP1	non-specific lipid-transfer protein P5	XM_002270934.2
VIT_07s0130g00030			-36,42	2,91	PDF2	defensin-like protein 1	XM_010654727.2

GeneID V1	S6	S24	T6	T24	GeneName	Product	GenBank Acc.
**Effector Triggered Immunity (ETI) - R Genes**							
VIT_13s0139g00210		-20,15			VIT_213s0139g00210	putative disease resistance RPP13-like protein 1	XR_787311.1
VIT_09s0002g05840		-16,21			RPS5_1	probabble disease resistance protein rps5-like	XP_002281195.1
VIT_15s0045g01020		-13,50			RPP8	putative disease resistance protein At1g50180	XM_010662983.1
VIT_13s0084g00510			6,92		VIT_213s0084g00510	PREDICTED: *Vitis vinifera* putative disease resistance RPP13-like protein 1	XM_019224396.1
VIT_12s0035g01280	-5,23		6,07		VIT_212s0035g01280	PREDICTED: *Vitis vinifera* disease resistance protein RFL1-like	XM_019223277.1
VIT_19s0027g01780			6,13		RPS5_2	probable disease resistance protein At1g12280	XM_010646742.1
VIT_13s0064g01780			6,58		VIT_213s0064g01780	putative disease resistance RPP13-like protein 1	XP_010659292.1
VIT_19s0027g00660			6,51		VIT_219s0027g00660	putative disease resistance protein RGA4	XM_010646688.1
VIT_19s0027g01580			5,81	-6,77	VIT_219s0027g01580	PREDICTED: *Vitis vinifera* probable disease resistance protein At1g12280-like	XM_019217064.1
**Imune response network**							
VIT_05s0077g00500	3,72	8,74			MYB108	*Vitis vinifera* R2R3 transcription factor MYB108-like protein 1	NM_001281062.1
VIT_03s0038g03410		-15,43			NAC036	PREDICTED: *Vitis vinifera* NAC domain-containing protein 35	XM_002281780.4
VIT_16s0039g01710			5,85	-4,58	MYB9	transcription factor MYB39	XM_003633958.2
VIT_08s0007g00410			-6,75		AS1	PREDICTED: *Vitis vinifera* transcription factor AS1	XM_002266391.3
VIT_06s0061g01270				-5,27	DME	PREDICTED: *Vitis vinifera* transcriptional activator DEMETER	XM_019220413.1
VIT_05s0062g00700		30,82			IAGLU_1	PREDICTED: *Vitis vinifera* crocetin glucosyltransferase, chloroplastic	XM_002263265.4
VIT_05s0062g00710	2,19	27,56			IAGLU_2	PREDICTED: *Vitis vinifera* crocetin glucosyltransferase, chloroplastic-like	XM_010652096.2
VIT_09s0002g04180		7,80			VIT_209s0002g04180	protein PLANT CADMIUM RESISTANCE 8	XM_002279124.3
VIT_11s0016g04750		10,94	-2,89		AKT1	potassium channel AKT1-like	XM_002281751.2
VIT_11s0016g00560		-12,77			ATPRX	peroxiredoxin Q	NM_001281040.1
VIT_10s0523g00010	-8,82	-12,81			VIT_210s0523g00010	heat shock protein 70 -interacting	
VIT_09s0054g00350		-12,72			VIT_209s0054g00350	atp gtp binding protein	
VIT_11s0037g00850		-17,24			VIT_211s0037g00850	PREDICTED: *Vitis vinifera* protein TSS	XM_003633119.2
VIT_04s0008g01530			6,52		HSP17II	17.3 kDa class II heat shock protein-like	XP_003631809.1
VIT_08s0007g00430			9,81		VIT_208s0007g00430	elicitor-responsive protein 1-like	XM_002270572.2
VIT_00s0371g00010	-2,60		6,49		CAD9	PREDICTED: *Vitis vinifera* probable mannitol dehydrogenase	XM_002271543.4
VIT_19s0090g01480			5,92		NHX3	sodium hydrogen exchanger 4-like	XM_002276777.2
VIT_11s0016g05850			6,60		VAMP711	vesicle-associated membrane	
VIT_19s0090g00400	-2,44		-5,61		VIT_219s0090g00400	NEP1-interacting protein-like 1	XM_010646150.1
VIT_00s0388g00070			5,17	-5,37	PGP9	PREDICTED: *Vitis vinifera* ABC transporter B family member 9	XM_019222449.1
VIT_18s0001g13200			-3,72	-9,07	CKX5	cytokinin dehydrogenase 5	XM_002280761.2
**Genome Machinery**							
VIT_13s0064g01260		-15,44			VIT_213s0064g01260	DNA-damage-repair/toleration protein DRT100-like precursor	NM_001281258.1
VIT_00s0274g00010			7,48		MSH3	PREDICTED: *Vitis vinifera* DNA mismatch repair protein MSH3-like	XM_019218309.1
VIT_01s0150g00390			-10,25		MCM2	DNA replication licensing factor MCM2	XM_002273639.2
VIT_03s0038g00300			-5,94		MCM4	DNA replication licensing factor MCM4	XM_002274498.2
VIT_06s0061g00600				-5,68	VIT_206s0061g00600	PREDICTED: *Vitis vinifera* protein NUCLEAR FUSION DEFECTIVE 4	XM_002273067.4

Table S6. Genes common to the ER-DEGs and DR-DEGs data sets.

Gene ID V1	S6	S24	T6	T24	GeneName	Product Name	GenBank Acc.
VIT_19s0015g00280		2,6	4,7		ATSPO11-1	PREDICTED: *Vitis vinifera* meiotic recombination protein SPO11-1	XM_010646261.2
VIT_09s0002g01350				3,6	GRF5	PREDICTED: *Vitis vinifera* growth-regulating factor 1	XM_010656138.2
VIT_00s0532g00020			-2,1		ATK1	PREDICTED: *Vitis vinifera* kinesin-like protein KIN-14C	XM_002265264.3
VIT_07s0129g00210		13,2	-2,7		BT4	BTB/POZ and TAZ domain-containing protein 4	XM_003632444.2
VIT_05s0020g02000				-4,5	SYD	PREDICTED: *Vitis vinifera* chromatin structure-remodeling complex protein SYD	XM_010651496.2
VIT_05s0077g00940			-2,4		PHYB	phytochrome B	XM_002278227.2
VIT_04s0023g00920				-4,1	DCL2	PREDICTED: *Vitis vinifera* endoribonuclease Dicer homolog 2	XM_019219502.1
VIT_14s0006g00640	2,0			-4,0	RGA1	PREDICTED: *Vitis vinifera* DELLA protein GAI (LOC100253268)	XM_002266231.4
VIT_08s0040g01440	1,8				PGK1	PREDICTED: *Vitis vinifera* phosphoglycerate kinase (LOC100243165)	XM_019221715.1
VIT_13s0067g01660		5,3			SYN1	sister chromatid cohesion 1 protein 1	XM_002273343.3
VIT_06s0004g05920			-2,5		PCNA2	PREDICTED: *Vitis vinifera* proliferating cell nuclear antigen-like	XM_002281054.3
VIT_03s0038g00300			-5,9		MCM4	DNA replication licensing factor MCM4	XM_002274498.2
VIT_01s0150g00390			-10,3		MCM2	DNA replication licensing factor MCM2	XM_002273639.2
VIT_18s0001g03080			-2,5		LYM1	lysM domain-containing GPI-anchored protein 1	XM_002285812.2
VIT_00s0227g00130				-4,1	VIT_200s0227g00130	PREDICTED: *Vitis vinifera* adenine DNA glycosylase	XM_002264991.4
VIT_04s0008g02570			5,1		RPA2	PREDICTED: *Vitis vinifera* replication protein A 32 kDa subunit A	XM_002282760.3
VIT_10s0003g03080			-3,4		MCM3	DNA replication licensing factor MCM3 homolog 2	XM_010657342.1
VIT_06s0061g01270				-5,3	DME	PREDICTED: *Vitis vinifera* transcriptional activator DEMETER	XM_019220413.1
VIT_16s0013g00310	-2,6				SUVH5	PREDICTED: *Vitis vinifera* histone-lysine N-methyltransferase, H3 lysine-9 specific SUVH5	XM_002277738.3
VIT_07s0005g01430			-3,0		PRL	PREDICTED: *Vitis vinifera* DNA replication licensing factor MCM7	XM_002276293.4
VIT_17s0000g09790	2,1				BT1	BTB/POZ and TAZ domain-containing protein 1-like	XM_002278156.3
VIT_11s0016g00340			-2,4		RAD51	DNA repair protein RAD51 homolog	XM_002273767.2
VIT_06s0061g01610	-2,5				TIL1	DNA polymerase epsilon catalytic subunit A	XM_002269884.3
VIT_16s0022g01820			-2,1		PLE	65-kDa microtubule-associated protein 3	XM_002267548.2
VIT_11s0016g05490			-3,0		ARP6	PREDICTED: *Vitis vinifera* actin-related protein 6	XM_010658136.2
